# Systematic optimization and evaluation of culture conditions for the construction of circulating tumor cell clusters using breast cancer cell lines

**DOI:** 10.1186/s12885-024-12214-9

**Published:** 2024-04-23

**Authors:** Jueyao Zou, Qiong Chen, Yong He, Yanhong Pan, Han Zhao, Junfeng Shi, Zhonghong Wei, Suyun Yu, Yang Zhao, Xin Han, Yin Lu, Wenxing Chen

**Affiliations:** 1 Jiangsu Key Laboratory for Pharmacology and Safety Evaluation of Chinese Materia Medica, School of Pharmacy, Nanjing University of Chinese Medicine, Nanjing, 210023 China; 2 Jiangsu Collaborative Innovation Center of Traditional Chinese Medicine (TCM) Prevention and Treatment of Tumor, Nanjing, China; 3 Jiangsu Joint International Research Laboratory of Chinese Medicine and Regenerative Medicine, Nanjing, China; 4 Department of Oncology, Nanjing First Hospital of Nanjing Medical University, Nanjing, China; 5 Department of Biochemistry and Molecular Biology, School of Medicine & Holistic Integrative Medicine, Nanjing University of Chinese Medicine, Nanjing, China; 6 Jiangsu Collaborative Innovation Center of Chinese Medicinal Resources Industrialization, Nanjing, China

**Keywords:** Circulating tumor cell clusters, Tumor metastasis, Culture methods, Molecular characteristics

## Abstract

**Background:**

Circulating tumor cell (CTC) clusters play a critical role in carcinoma metastasis. However, the rarity of CTC clusters and the limitations of capture techniques have retarded the research progress. In vitro CTC clusters model can help to further understand the biological properties of CTC clusters and their clinical significance. Therefore, it is necessary to establish reliable in vitro methodological models to form CTC clusters whose biological characteristics are very similar to clinical CTC clusters.

**Methods:**

The assays of immunofluorescence, transmission electron microscopy, EdU incorporation, cell adhension and microfluidic chips were used. The experimental metastasis model in mice was used.

**Results:**

We systematically optimized the culture methods to form in vitro CTC clusters model, and more importantly, evaluated it with reference to the biological capabilities of reported clinical CTC clusters. In vitro CTC clusters exhibited a high degree of similarity to the reported pathological characteristics of CTC clusters isolated from patients at different stages of tumor metastasis, including the appearance morphology, size, adhesive and tight junctions-associated proteins, and other indicators of CTC clusters. Furthermore, in vivo experiments also demonstrated that the CTC clusters had an enhanced ability to grow and metastasize compared to single CTC.

**Conclusions:**

The study provides a reliable model to help to obtain comparatively stable and qualified CTC clusters in vitro*,* propelling the studies on tumor metastasis.

**Supplementary Information:**

The online version contains supplementary material available at 10.1186/s12885-024-12214-9.

## Introduction

Metastasis is a major cause of death in cancer patients, with tumor cells spreading through the bloodstream to form secondary tumors distantly [1]. Circulating tumor cell (CTC) clusters (composed of two or more tumor cells) have a metastatic advantage over individual CTCs [2, 3], which makes them a very clinically relevant population of cells to be targeted. Targeting CTC clusters is considered to be an effective therapeutic strategy [4, 5]. However, the underlying mechanisms of the high metastatic capacity of CTC clusters remain to be investigated. The rare number of CTC clusters and the difficulty of capturing them have prevented researchers from obtaining sufficient numbers of CTC clusters for downstream analysis [4], resulting in a very slow development of the molecular biology of CTC clusters. The current research mainly focuses on exploring the diagnostic, prognostic, and clinical significance of CTC clusters [6]. The heterogeneity of CTC clusters also makes it difficult to uncover universal scientific phenomena in terms of metastasis [7, 8]. CTC permanent cell lines appear to be difficult to be cultured and have a relatively low success rate, resulting in the small number available. In addition, the final molecular features of CTC clusters after long-term culture no longer represent the original tumor phenotype, so the availability of CTC cell lines is often questioned [9].

Due to this restricted condition, in vitro CTC cluster models generated using mature cancer cell lines are being used with increased frequency. Researchers can use such in vitro models for exploring the biological characteristics of CTC clusters [10–12] and assessing the pharmacological effects of cluster dissociation agents [13–16]. Nowadays, in vitro CTC cluster models have become indispensable tools to help us understand the mechanisms of high metastatic potential of CTC clusters. However, the construction of in vitro models for CTC clusters is still at an immature stage and their plausibility is frequently overlooked. The main issue is that the culture conditions for in vitro CTC cluster models are diverse and mainly rely on the researchers' own experience without theoretical basis, which leads to a high biological variability for the in vitro models. For example, the utilization of trypsin to obtain multicellular structures by virtue of incomplete digestion only considers the morphological similarity of the cell clusters [10], whereas the other phenotypes of the cells are almost identical to those of the adherent cells, such as expression of adhesion proteins by the cells as well as the activation of metastasis-associated signaling pathways within the cells. Using ultralow-attach (ULA) plate to grow cells in suspension allows cells to spontaneously aggregate into multicellular bodies [17]. However, it is performed in a static environment and ignores the fluid shear force that affects the aggregation of tumor cells. Clearly, various current methods for constructing in vitro models of CTC clusters are controversial. When tumor cells enter the circulatory system from the primary site, they have short survival time [18, 19], but their molecular characteristics tend to alter much earlier [20, 21]. Therefore, transient suspension culture of cancer cell lines and the use of conventional culture conditions make it difficult for CTC clusters in vitro models to produce cell phenotypes similar to those of clinical CTC clusters. The alteration of cell phenotype is related to the suspension state of the cells, the flow rate of the liquid, the oxygen concentration, and other factors, so there is an urgent need for a longer induction time and richer induction conditions. Therefore, it is necessary to establish relatively reasonable culture conditions for in vitro CTC cluster models and thus improve their biological similarity.

The scientific validity and reasonableness of in vitro CTC cluster models can be evaluated on the basis of their similarity to real CTC clusters. Whether the experimental model adequately restores the original molecular characteristics of CTC clusters will directly affect the reliability of the experimental results, while an inappropriate cellular model can lead to the omission of important molecular information. It emphasizes that evaluation of the model cannot be omitted. Since the biological properties of CTC clusters have been partially revealed, such as epithelial/mesenchymal hybrid phenotype (E/M hybrid phenotype) [22–24], high expression of adhesion proteins [11, 25], and deformability [26], the research advancements have enabled the evaluation of similarity between in vitro CTC cluster models and clinical CTC clusters in terms of molecular characteristics and cellular behavior.

In this study, we took consideration of the effect of the physiological environment of CTC clusters in the circulatory system on their molecular characteristics, including the hypoxic environment, blood shear stress and suspended state. We aimed to stimulate in vitro CTC clusters using breast cancer cell lines. More critically, we highlighted the biosimilarity of in vitro CTC clusters with clinical real cell clusters in the aspect of cellular behavior and molecular features at various stages of metastasis including invasion, intravasation, survival in the circulatory system, extravasation and colonization at remote site.

## Materials and methods

### Cell lines

Estrogen receptor positive (ER +) human-derived breast cancer MCF-7(RRID: CVCL_0031), triple negative breast cancer (TNBC) human-derived breast cancer MDA-MB-231(RRID: CVCL_0062), and highly metastatic mouse-derived 4T1 cell lines were grown at 37℃, 5% CO_2_ in DMEM medium supplemented with 10% fetal bovine serum and 1X penicillin/streptomycin. HUVEC cell line (RRID: CVCL_9Q53) was grown at 37℃, 5% CO_2_ in RPMI 1640 medium supplemented with 10% fetal bovine serum and 1X penicillin/streptomycin. All cell lines were from American Type Culture Collection, and were tested regularly for mycoplasma contamination.

To create single cells, the adherent cells were fully digested into individual cells with trypsin. To create CTC clusters, the cells were added to the culture flask at a density of 1 × 10^5^ per milliliter and incubated on a shaker (50 rpm/min). Meanwhile, the oxygen and FBS concentrations decrease to 5% and 2.5%, respectively. 10 ng/mL epidermal growth factor (EGF) (MedChemExpress, HY-P7109, USA) and 10 ng/mL basic fibroblast growth factor (bFGF) (MedChemExpress, HY-P7004, USA)were added as nutrition sources.

### Tail vein pulmonary metastasis model

4 ~ 6 week old female BALB/c mice were provided by Shanghai Slaughter Laboratory Animal Co.LTD. To establish a bioluminescence-based lung metastasis model, luciferase-expressing 4T1 cells (1.5 × 10^6^ cells/0.15 mL) were prepared as single cells and cell clusters and injected into the lateral tail vein of mice. Before injection, a portion of the cell cluster group was digested with trypsin into single cells and counted to ensure that the total number of cells injected into the tail vein of both the single cell group and the cell cluster group was consistent. On day 1, 2, 7 and 14, mice were respestively anesthetized and injected with 150 mg/kg D-fluorescein in the fundus venous plexus. Lung metastases were monitored by bioluminescence imaging using the IVIS spectroscopy system (Perkin Elmer, USA). Body weight was measured every three days. Two weeks after modeling, mice were sacrificed, and lung tissues were harvested for further hematoxylin–eosin (H&E) staining and histological examination.

### Chip design and fabrication

The microfluidic chip pattern is based on the design of the paper [27]. The Cluster-Chip captures CTC clusters by relying on the strength of cell–cell junctions as clusters flow at physiological speed through a set of triangular pillars. Three pillars make up the basic unit of the chip; two form a narrowing channel that funnels the cells into an opening, where the edge of the third pillar is positioned to bifurcate the laminar flow. As blood flows, single blood and tumor cells divert to one of the two streamlines at the bifurcation and pass through the 12 µm × 100 µm opening. In contrast, CTC clusters are held at the edge of the bifurcating pillar. The microfluidic device was fabricated according to standard photolithography and soft lithography procedures. The negative photoresist SU8-3025 (MicroChem, USA) pattern on the silicon wafer was fabricated with a photomask. The silicon wafer was then silanized with trimethylchlorosilane (Thermo Scientific, USA) to facilitate PDMS mold release PDMS prepolymer (Dow Corning, USA) was poured onto the silicon wafer and cured at 80°C for 1 h. Holes were punched in the PDMS, and oxygen plasma treatment was used to chemically bond the PDMS mold to a glass slide.

### Microfluidic chips capture CTC clusters

After preparation of the 4T1 cell cluster hematogenous metastasis model or the advanced 4T1 cell orthotopic tumor model, mice were anesthetized using isoflurane (RWD, R510-22–10, China) at a concentration of 2.5% until the lack of response to stimulation of the mice's toes was the mark of successful anesthesia, and a small amount of blood was collected by cardiac puncture and collected in EDTA anticoagulant tubes (BD, 367862, USA), and then the mice were sacrificed. The blood was diluted 1:1 with phosphate buffer saline (PBS) and used for microfluidic microarrays (provided by Prof. Xin Han of Nanjing University of Chinese Medicine). The flow rate of blood through the microchip was adjusted to 10 mL/h, then residual blood cells were washed with PBS at a rate of 10 mL/h. CTC clusters were identified by immunocytochemistry. Cells were fixed in 4% paraformaldehyde for 30 min and then blocked with 1% bovine serum albumin for 1 h. Immunohistochemical staining was performed with a combination of Pan-Cytokeratin (Santa Cruz Biotechnology, sc-8018, mouse monoclonal) and CD45 (Santa Cruz Biotechnology, sc-1178, mouse monoclonal) antibodies. Subsequently, DAPI was used to stain for nuclei. CTC clusters were identified as (1) Pan-Cytokeratin protein positive; (2) CD45 negative; (3) DAPI positive; and (4) aggregates of two or more cells. Alternatively, cells expressing GFP or mCherry were captured on a microarray, washed with PBS, and imaged directly. Imaging was performed with a Zeiss Axio vert A1 microscope.

### Immunofluorescence

Cell clusters were prepared by the method described previously (2.1), and attached to adherent slides using a cell smear centrifuge (500 g/min, 3 min) to facilitate subsequent cell immunofluorescence manipulations. After the cells adhered to the slides, they were fixed in 4% paraformaldehyde and permeabilized with PBS containing 0.1% Triton X-100. After blocking with 1% BSA for 1 h, the cells were incubated with primary antibodies overnight. Then, the cells (adherent cells: 1 × 10^6^ cells; cell clusters: 1 × 10^5^ cells) were coincubated with Goat Anti-Mouse IgG H&L (FITC) (Abcam, ab6785) and Goat Anti-Rabbit IgG H&L (TRITC) (Abcam, ab6718) secondary antibodies, respectively. Images were captured using a Zeiss Axio vert A1 microscope. The primary antibodies involved were as follows: plakoglobin (PG) (Proteintech, 27,872–1-AP, rabbit polyclonal), ZO-1 (Proteintech, 21,773–1-AP, rabbit polyclonal), Carcinoembryonic Antigen Related Cell Adhesion Molecule 6 (CEACAM6) (ABclonal, A5971, rabbit polyclonal), vimentin (Santa Cruz Biotechnology, sc-6260, mouse monoclonal), Programmed Cell Death-Ligand 1 (PD-L1) (Proteintech, 66,248–1-lg, mouse monoclonal), claudin 5 (Abcam, ab15106, rabbit polyclonal).

### Transmission electron microscopy

Tumor cell clusters were washed by DPBS (Gibco, C14190500BT), then fixed in 2.5% glutaraldehyde (LEAGENE, DF0156) at 4℃ for at least 16 h. Samples were then sent to the Research Dog Instrument Testing Platform and visualized using a JEM1400 transmission electron microscope operated at 120 kV.

### Western blotting

Cells were lysed in RIPA Lysis Buffer (Beyotime, P0013B) containing protease inhibitors and phosphatase inhibitors. Protein concentration was measured by a BCA kit (Beyotime, P0012S). 1.5 × 10^6^ cells per group were lysed and about 20 μg proteins were loaded for SDS-PAGE. After proteins were transferred to a PVDF membrane, skim milk (5%) was used for blocking and then stained with the following antibodies: Plakoglobin, ZO-1, PD-L1, β-actin (Proteintech, 66009–1-Ig, mouse monoclonal), CEACAM6 (ABclonal), Integrin β1(Cell Signaling Technology, 4706P, rabbit polyclonal), Integrin β5 (Cell Signaling Technology, 4708P, rabbit polyclonal), vimentin (Santa Cruz Biotechnology), Anti-HIF-1α (Abcam, ab15106, rabbit polyclonal), GAPDH (Bioworld, AP0063, rabbit polyclonal). Goat anti-Rabbit IgG(H&L)-HRP (Bioworld, BS13278) and Goat anti-Mouse IgG(H&L)-HRP (Bioworld, BS12478) were used as the second antibodies. The blots were scanned by Gel Imaging System (Bio-Rad, ChemiDoc XRS + , USA) with ECL western blotting kit. Protein expressions were quantified using Image lab software (Bio-Rad, ChemiDoc™ XRS + , USA).

### Limiting dilution assay

A limiting dilution assay (LDA) was performed on MCF-7 adherent cells and suspension cells. Cells were seeded in 6-well plates at concentrations of 2000 cells per well and cultured for 7 days. Suspension cells were cultured under the non-adherent culture conditions described above. Sphere formation was determined as spheres with diameters ≥ 50 µm. Significant statistical differences in samples were determined by a 95% confidence interval (CI).

### Time-lapse imaging for migration and outgrowth analysis

Single or clustered cells were plated in Matrigel gels (Corning, 354234, USA). Differential Interference Contrast (DIC) images were captured hourly using an IncuCyte Zoom (Essen Bioscience, USA) at 40** × **magnification. Exposure times were < 30 ms for DIC.

### EdU incorporation assay

Cell proliferation was determined by the incorporation of 5-ethynyl-20-deoxyuridine (EdU). EdU cell proliferation staining was performed using an EdU kit (Beyotime, C0075S). Briefly, MCF-7, MDA-MB-231, and 4T1 cells (8 × 10^5^ cells/well) were seeded in 6-well plates and cultured for 24 h. Subsequently, cells were treated with EdU for 2 h, fixed with 4% paraformaldehyde for 15 min, and permeated with 0.3% Triton X-100 for another 15 min. The cells were incubated with the Click Reaction Mixture for 30 min at room temperature in a dark place and then incubated with DAPI for 10 min.

### Cell adhesion assay

The 96-well plates were seeded with 8 × 10^4^ MCF-7 cells or HUVEC cells and incubated for 24 h. The next day, 2 × 10^4^ MCF-7-GFP adhesion cells or suspension cells were seeded into the well plate, incubated for 30 min, and inadherent cells were removed by washing with PBS 3 times. The number of GFP-labeled adherent cells was observed under a fluorescent microscope, and 5 fields of view were taken for each group. The GFP-labeled adherent cells were qualified by Image J software and the adhesion efficiency was calculated.

### RNA extraction and real-time PCR

Total RNA was extracted using TRIzol reagent (Vazyme, R411-01). For synthesis of cDNA, HiScript®R II Q RT SuperMix for qPCR (Vazyme, R223-01) was used according to the manufacturer's instructions. Quantitative real-time PCR was performed by using a ChamQ™ SYBR R® qPCR Master Mix (High ROX Premixed) (Vazyme, Q331-02) according to the manufacturer's instructions. GAPDH gene was used as an internal standard gene and the 2 − ∆∆CT method was utilized to quantitatively analyse the data. The primer sequences are shown below: human GAPDH Forward: ACGGATTTGGTCGTATTGGG, Reverse: GGGATCTCGCTCCTGGAAG; human JUP Forward: TCGCCATCTTCAAGTCGGG, Reverse: AGGGGCACCATCTTTTGCAG;

### Clinical samples

A total of four patients were included in this study, with the tumor tissue type of breast cancer and the subtype of estrogen receptor-positive (HR +)/HER2-. All patients had stage III or IV tumors. The treatment regimen consisted of cisplatin in combination with chemotherapy. Blood samples were collected in the 1st month after treatment, and the sampling condition was fasting morning collection. CTC isolation technique was based on microfluidic chip-based method.

### Statistical analysis

Each experiment was repeated at least three times and all data are presented as mean ± SD. The statistical analyses were conducted with GraphPad Prism 6 software. Pairwise comparisons were made using a two-sample t-test (parametric data). Comparisons between multiple groups were made by one-way analysis of variance (ANOVA) followed by Bonferroni multiple comparison test. *P* < 0.05 was considered statistically significant (* *P* < 0.05; ** *P* < 0.01; *** *P* < 0.001).

## Results

### Constructing CTC clusters in vitro and assessing its molecular characterization

**Fig. 1:**
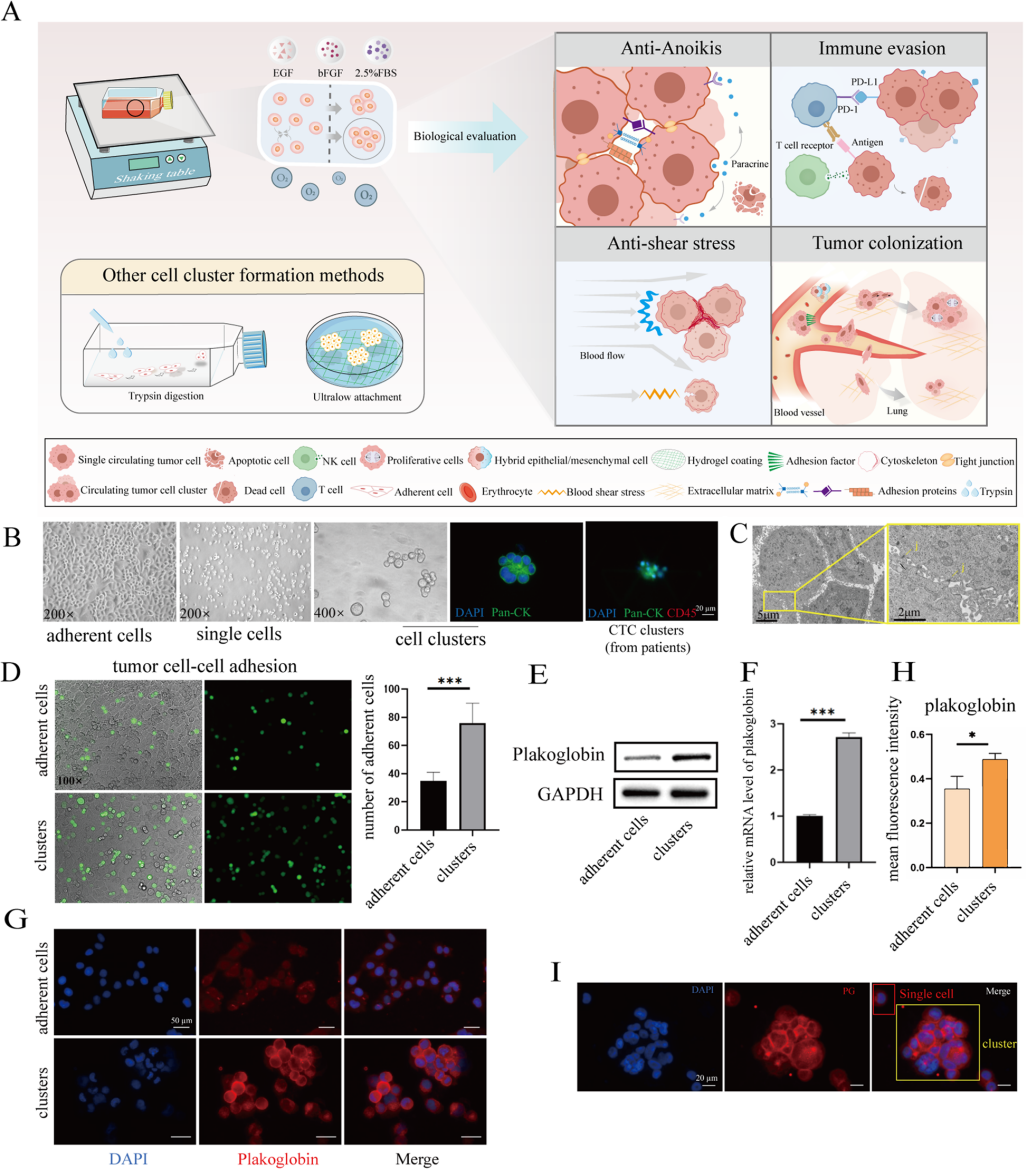
Construction of in vitro model of CTC clusters and preliminary evaluation of its molecular characteristics. **A** Schematic of the experimental for cell clusters formation and evaluation. **B** Representative images of the appearance of MCF-7 cell clusters and CTC clusters from patients. Pan-Cytokeratin (Pan-CK) (green), DAPI (nuclei, blue). **C** Transmission Electron Microscope (TEM) images of MCF-7 cell clusters. J, cell–cell junction. **D** Left: tumor cell–cell adhesion test. The lower layer is MCF-7 cells, and the upper layer is MCF-7-GFP cells (green). Right: the quantitated results, *n* = 5, ***P < 0.001. **E** Western blot analysis of the protein levels of plakoglobin in MCF-7 adherent cells and cell clusters. **F** Q-PCR analysis of the mRNA levels of plakoglobin in MCF-7 adherent cells and cell clusters. *n* = 3, ***P < 0.001. **G** Adhesion molecules localization. MCF-7 adherent cells and cell clusters were stained for DAPI (nuclei, blue) and plakoglobin (red) by immunofluorescence assay. The quantitated results of plakoglobin are shown in (**H**), *n* = 5, *P < 0.05. **I** Immunofluorescence analysis of the protein levels difference of plakoglobin in MCF-7 single cells and cell clusters. DAPI (nuclei, blue), plakoglobin (red). Two tail student's t-test analysis was used to compare the statistical difference between indicated two groups in (**D**), (**F**), and (**H**)

In order to enable the CTC formation environment as similar as possible to the physiological state, we took into account the physiological conditions such as blood shear stress, nutrient conditions, and oxygen conditions that CTC clusters need to face in the circulation, and constructed a cell cluster model that is more suitable for in-depth studies. Therefore, we used a shaker to simulate blood shear stress and provided a nutrient environment with low oxygen, as well as low levels of serum and growth factors, and finally assessed the similarity of the biological behavior of the cell clusters to CTC clusters during metastasis including resistance to anoikis, immune escape, resistance to blood shear, and colonization of the tumor metastases (Fig. 1A). The concrete culture conditions are shown in Table 1.

**Table 1:** Comparison of different culture conditions for the construction of cell clusters using cell lines

Culture conditions in this article	Culture conditions in references	Disadvantages	References
2.5% fetal bovine serum Hypoxia (5% O_2_) 10 ng/mL epidermal growth factor (EGF) 10 ng/mL basic fibroblast growth factor (bFGF) Shaking Table (50 rpm/min) Cells grown in suspension	Incubated with trypsin; 10% fetal bovine serum; normoxia	Loose structure, Low adhesion	[10]
	Serum-free medium (including 20 ng/mL EGF and 20 ng/mL bFGF); normoxia	Loose structure, Low adhesion, Expensive	[3]
	Ultra-Low attachment cell culture flasks; 10% fetal bovine serum; normoxia	Static cultivation, Expensive	[17]
	The Poly-HEMA- treated six-well plates; 10% fetal bovine serum; normoxia	Static cultivation, Expensive	[15, 28]
	Shaking table-based method (60 rpm/min); 10% fetal bovine serum; normoxia	Not close to the real environment	[29]

By virtue of this methodology, we successfully obtained the tumor cell clusters for MCF-7, MDA-MB-231 and 4T1 cells, which were very similar to the in vivo clusters (identified as Pan-CK-positive and CD45-negative) in size and morphology. They are mostly composed of 2 to 40 cells with either a spherical or chained morphology and expressed Pan-CK (Fig. 1B & Figure S1A, Figure S3).

To confirm the reliability of our method, we firstly evaluated the similarity of the intercellular junctions. Observation of the intercellular microstructure of MCF-7 and 4T1 cell clusters by transmission electron microscopy (TEM) confirmed the presence of both tight junctions and adherens junctions in the cultured cell clusters (Fig. 1C & Figure S1B). Next, we examined the cell adhesion capacity in MCF-7 cell clusters by cell adhesion force assays, and the data showed that the adhesion capacity was significantly upregulated in cell clusters compared to that of the same cell line in the adherent state (Fig. 1D). The above results indicated that the cultured cell clusters according to our optimized methods presented a stable multicellular structure, which was consistent with CTC clusters in vivo, and could maintain the morphology of cell clusters in the blood.

The relationships between adhesion capacity and the expression of adhesion proteins of cell clusters built established upon our condition were also examined. Plakoglobin (PG), the key protein for CTC clusters to maintain a multicellular structure [25, 30], was examined in MDA-MB-231, MCF-7, and 4T1 cell clusters, and its expression was significantly up-regulated in cell clusters compared to adherent cells (Fig. 1E & Figure S1C). We also found an up-regulation in the mRNA expression of PG in the cell clusters (Fig. 1F). The results of cellular immunofluorescence were consistent with that of western blot analysis (Fig. 1H), and in the cell clusters, PG was enriched mainly on the cell membranes and was highly expressed at the cell junctions (Fig. 1G & Figure S1D). Moreover, the expression of PG was dramatically higher in cell clusters than in the single cells (Fig. 1I). These results suggested that PG was highly expressed in our cultured cell clusters, especially in the contact region between cells, implying its contribution to the interconnection between cells.

**Fig. 2:**
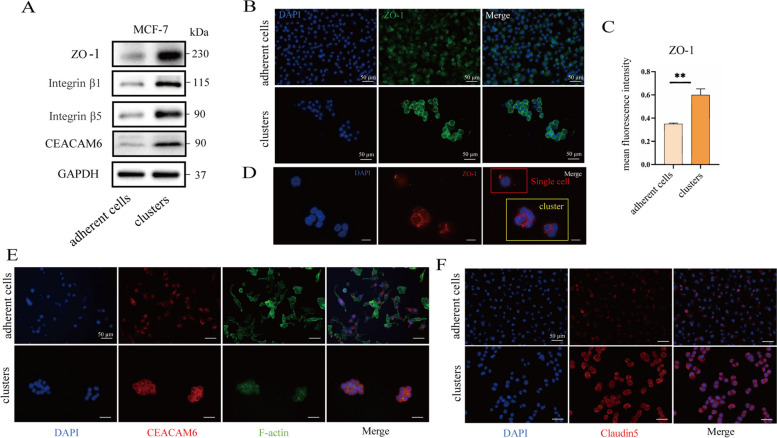
Other important adhesion proteins up-regulated in cell clusters. **A** Western blot analysis of the protein levels of ZO-1, integrin β1, integrin β5, CEACAM6 in MCF-7 cells. **B** MCF-7 adherent cells (1 × 10^6^ cells) and cell clusters (1 × 10^5^ cells) were stained for DAPI (nuclei, blue) and ZO-1 (green) by immunofluorescence assay. The quantitated results are shown in (**C**), *n* = 5, **P < 0.01. Two tail student's t-test analysis was used to compare the statistical difference between indicated two groups. **D** Immunofluorescence analysis of the protein levels difference of ZO-1 in 4T1 single cells and cell clusters. DAPI (nuclei, blue), ZO-1 (red). Scale bars, 20 µm. MCF-7 adherent cells (1 × 10^6^ cells) and cell clusters (1 × 10^5^ cells) were stained for (**E**) DAPI (nuclei, blue), CEACAM 6 (red), f-actin (green) and (**F**) claudin5 (red) by immunofluorescence assay

We also examined the expression of other adhesion proteins in the cell clusters, including ZO-1, carcinoembryonic antigen related cell adhesion molecule 6 (CEACAM6), integrin β5, integrin β1, etc. It was found that ZO-1, CEACAM6, integrin β5, and integrin β1 were significantly upregulated in the cell clusters compared to that in the adherent cells, and were highly expressed on the cell membranes (Fig. 2A-C, E, F & Figure S1E). Further studies revealed that ZO-1 expression was higher at cell junctions in clusters than in single cells (Fig. 2D), suggesting that cells in cell clusters were connected to each other by tight junctions and multiple adhesion protein-mediated adhesion junctions.

These results indicated that the cell cluster model possessed partial similarity to the spontaneous production of CTC clusters in patients in vivo, both in terms of cluster appearance as well as the properties of high expression of multiple adhesion proteins.

### Advantages of the in vitro CTC clusters culture method over other methods

**Fig. 3:**
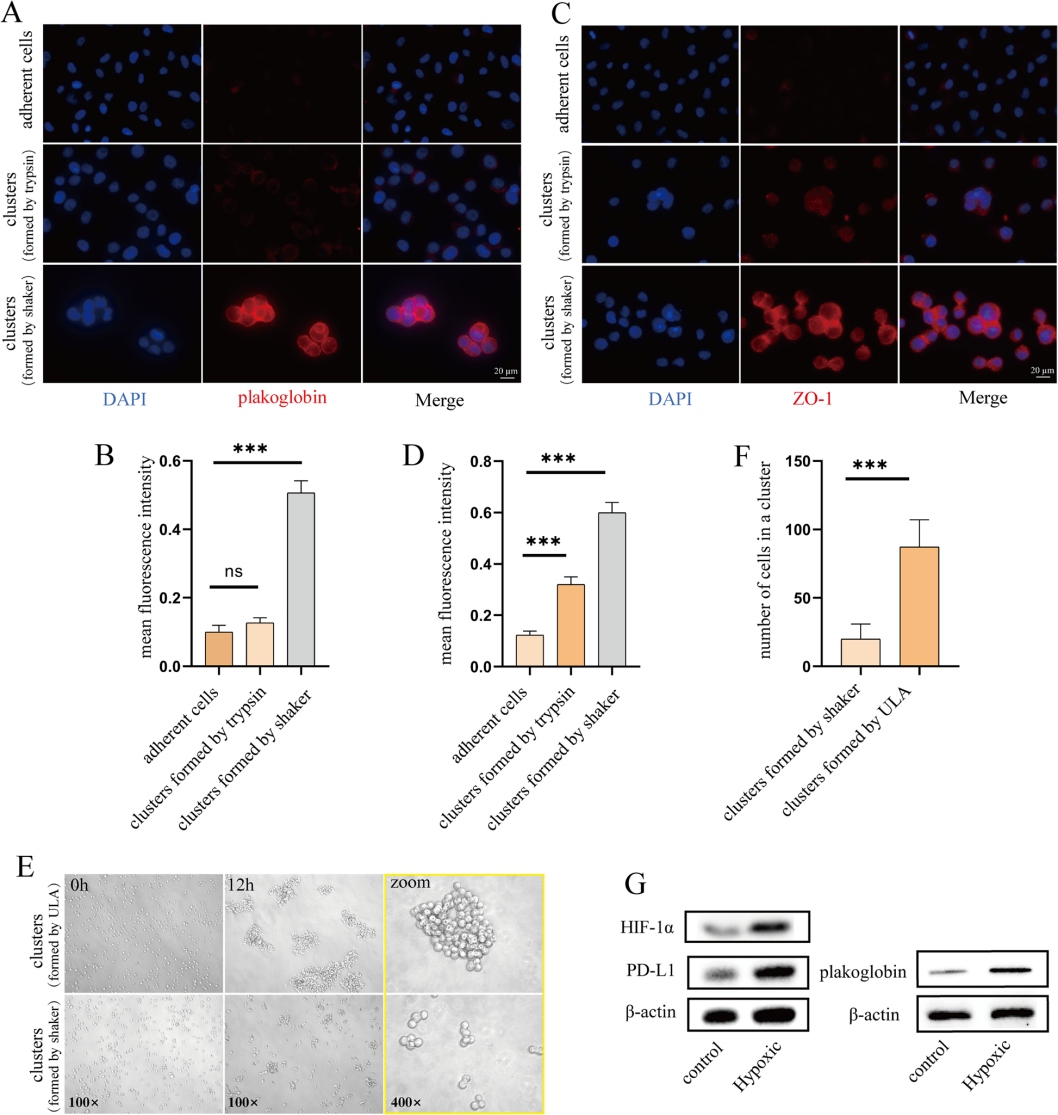
Advantages of the in vitro CTC cluster model. **A** Analysis of plakoglobin expression in different cell clusters by immunofluorescence. DAPI (nuclei, blue), Plakoglobin (red). The quantitated results are shown in (**B**), *n* = 5, ***P < 0.001. **C** Analysis of ZO-1 expression in different cell clusters by immunofluorescence. DAPI (nuclei, blue), ZO-1(red). The quantitated results are shown in (**D**), *n* = 5, ***P < 0.001. **E** Representative images of the appearance of cell clusters form ultralow-attach (ULA) plate and shaker. The quantitated results are shown in (**F**), *n* = 10, ***P < 0.001. **G** Western blot analysis of the effect of hypoxia culture on plakoglobin, PD-L1 and HIF-1α expression

To highlight the advantages of our cell cluster model, we compared it with other methods for generating cell clusters on a case-by-case basis. Compared with the trypsin digestion method, our cell clusters presented up-regulated adhesion proteins and a firm cell cluster structure, whereas the adhesion protein (PG and ZO-1) expression level of the clusters obtained by the trypsin method was almost identical to that of the adherent cells, and the clusters were easily separated into individual cells (Fig. 3A, B & C, D).

Compared with the method of ultra-low adsorption pore plates, the cell clusters cultured in ultra-low adsorption plates tended to become huge lamellar cell aggregates, and the size and morphology of the cell clusters were different from the clinical CTC clusters. In contrast, our method places the cells on a shaking bed that allows the cells to be in a fluid state and the cell clusters to grow in a decentralized form of moderate size (Fig. 3E, F). Additionally, the shaking generated by the shaking bed can also to some extent mimic blood shear stress, inducing cells closer to their physiological state by virtue of mechanical stimulation.

Compared to normoxic environments, hypoxia leads to an up-regulation of intercellular junctions and promotes intravascular aggregation of CTC clusters. Moreover, CTC clusters in hypoxic conditions have a higher capacity for metastasis [31]. Therefore, we chose 5% oxygen to simulate the hypoxic environment in the circulation. It was found that the hypoxic environment increased the expression of hypoxia-inducible factor-1α (HIF-1α) in the cell clusters compared to normoxic culture, along with the up-regulation of PG expression and immune checkpoint programmed cell death-ligand 1 (PD-L1) expression (Fig. 3G). High expression of these proteins may be more favorable for the survival of cell clusters within the bloodstream.

In conclusion, the conditions of cell cluster formation in our model take into account the physiological environment in the blood. In addition to this, the cell phenotypes undergo various changes following prolonged suspension domestication induction, including up-regulated adhesion proteins, and a series of changes resulting from the epithelial-mesenchymal transition (EMT) state and blood shear force.

### Viability of CTC clusters in the circulation system

**Fig. 4:**
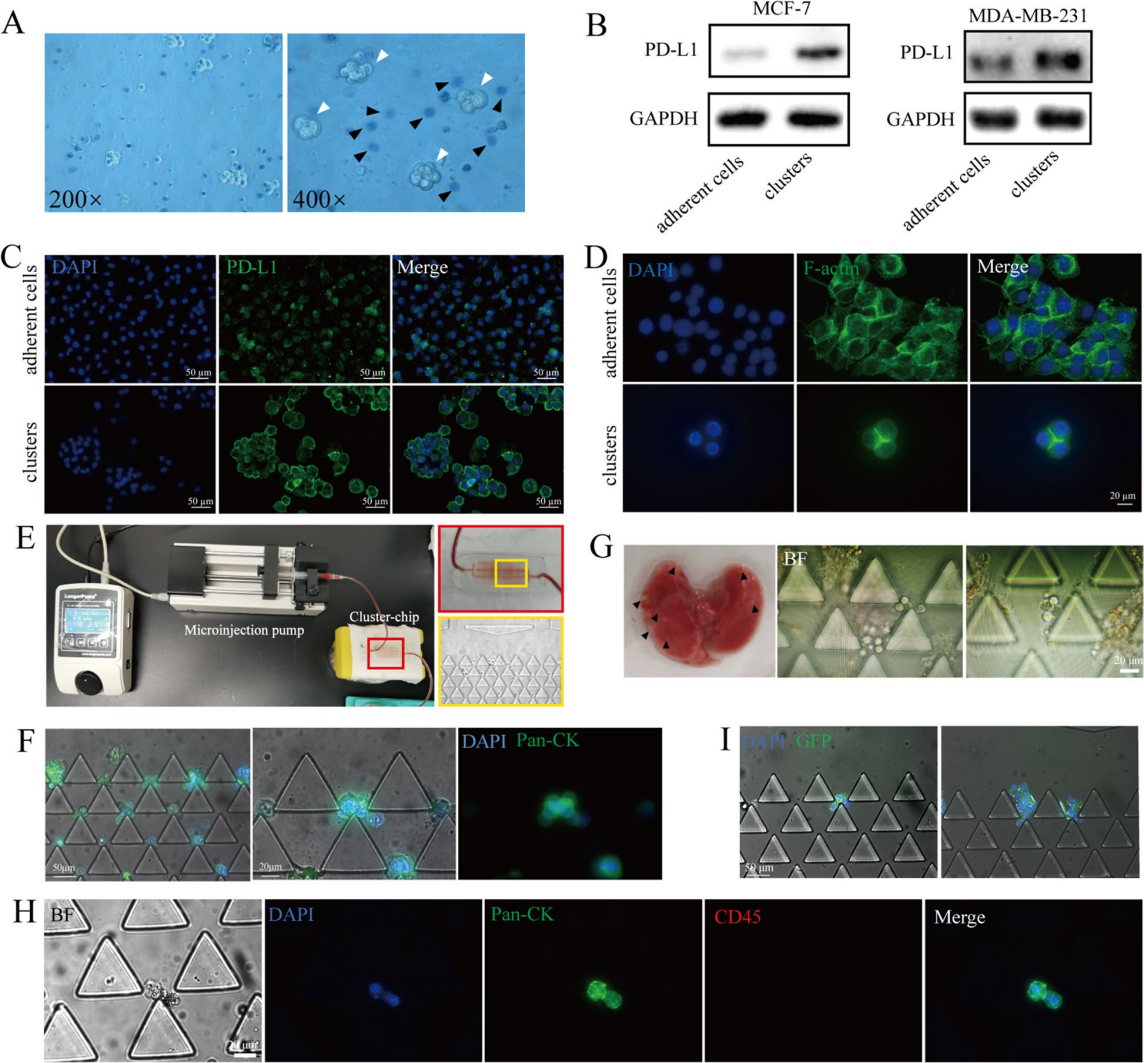
Viability of cell clusters in the circulatory system. **A** Trypan blue staining of single MCF-7 cells and cell clusters. The black arrow indicates single cell, whereas the white arrows point to cell cluster. **B** Western blot analysis of the protein levels of PD-L1 in MCF-7 and MDA-MB-231 cells. **C** Immunofluorescence analysis of the protein levels of PD-L1 in MDA-MB-231 cells. DAPI (nuclei, blue), PD-L1 (green). **D** Immunofluorescence analysis of the cytoskeleton localization in 4T1 cells. DAPI (nuclei, blue), F-actin (green). **E** Design and operation of the cluster-chip. **F** The cluster-chip captures MCF-7 cell clusters. Scale bars,20/50 µm. Pan-CK (green), DAPI (nuclei, blue). **G** Left: lung of mice with breast cancer in situ, the black arrow points to lung metastases. Right: the cluster-chip captures CTC clusters in bright field. Blood was collected from a mouse model of breast cancer in situ. Scale bars,20 µm. **H** Representative images of spontaneously shed CTC cluster in vivo. Pan-CK (green), CD45 (red) and DAPI (nuclei, blue). Scale bars, 20 µm. **I** The cluster-chip captures CTC clusters. Blood was collected three days after 4T1-GFP cell clusters were injected into the tail vein of mice. DAPI (nuclei, blue), GFP (green). Scale bars, 50 µm

Anoikis is the most important cause of tumor cell death after entering the bloodstream [32]. CTC clusters can minimize the occurrence of anoikis through intercellular junctions. The above results showed the presence of tight junctions and high expression of adhesion proteins in the cell clusters, which implied that the cell clusters established in our conditions were anti-apoptotic. To confirm this speculation, cell clusters and individual cells were stained with Trypan Blue, which showed that cells in clusters were more likely to maintain cell membrane integrity compared to individual cells (Fig. 4A). In addition, the CEACAM6 correlated with the ability to resist anoikis [33] was highly expressed in cell clusters compared to adherent cells (Fig. 2E). Both suggested that cell clusters were more resistant to anoikis than individual cells.

The strong immune evasion ability is another driving force for the survival of CTC clusters in the circulation [34, 35]. PD-L1 plays an important role in evading the killing of tumor cells by T cells. To this end, we demonstrated that its expression was significantly up-regulated in cell clusters compared to adherent cells and mainly located at the cell membrane, which may explain the ability of CTC clusters to evade immune attack in the bloodstream. (Fig. 4B-C & Figure S2A).

Shear in the blood can be lethal to cells, and it has been shown that breast cancer cells can adapt to blood shear and produce increased EMT and cancer stem cells (CSCs) [36, 37]. We then further examined the adaptive behavior of cell clusters in response to blood shear. We found that the cytoskeleton of cell clusters was altered under the stimulation of fluid flow, and cell microfilaments in cell clusters were redistributed, mainly concentrated at intercellular junctions (Fig. 4D), which facilitated the maintenance of a stable multicellular structure of cell clusters in the flowing state, thus slowing down the impulse force caused by blood flow. The above results indicated that cell clusters were able to resist and respond adaptively to the damage caused by blood shear forces.

To examine the survival of the cell clusters in vivo, we studied the cell clusters in vivo with the aid of microfluidic chips (Fig. 4E). First, we injected cell clusters into the microfluidic chip to verify the capture capacity of the chip and the stability of the clusters during capture. The results indicated that the microfluidic chip could capture larger single cells as well as clusters of more than two cells, and the clusters could maintain a multicellular structure during capture (Fig. 4F & Figure S2B). Next, we used the microarray to capture CTC clusters generated in mice with breast cancer in situ, and the experimental results showed that the microarray could capture rare CTC clusters (Fig. 4G, H).

To examine whether the cell clusters could survive and maintain the multicellular structure after entering the circulation, we injected the cell clusters into the tail vein of mice and took blood at intervals for the microarray assay. It was found that the clusters could be captured by the microarray and survive in the circulation (Fig. 4I). The above results suggested that the CTC cluster model exhibited survival advantages in blood and was a reliable model that could replace spontaneous CTC clusters in vivo for downstream studies.

### Assessment of the invasive capacity of CTC clusters in vitro

**Fig. 5:**
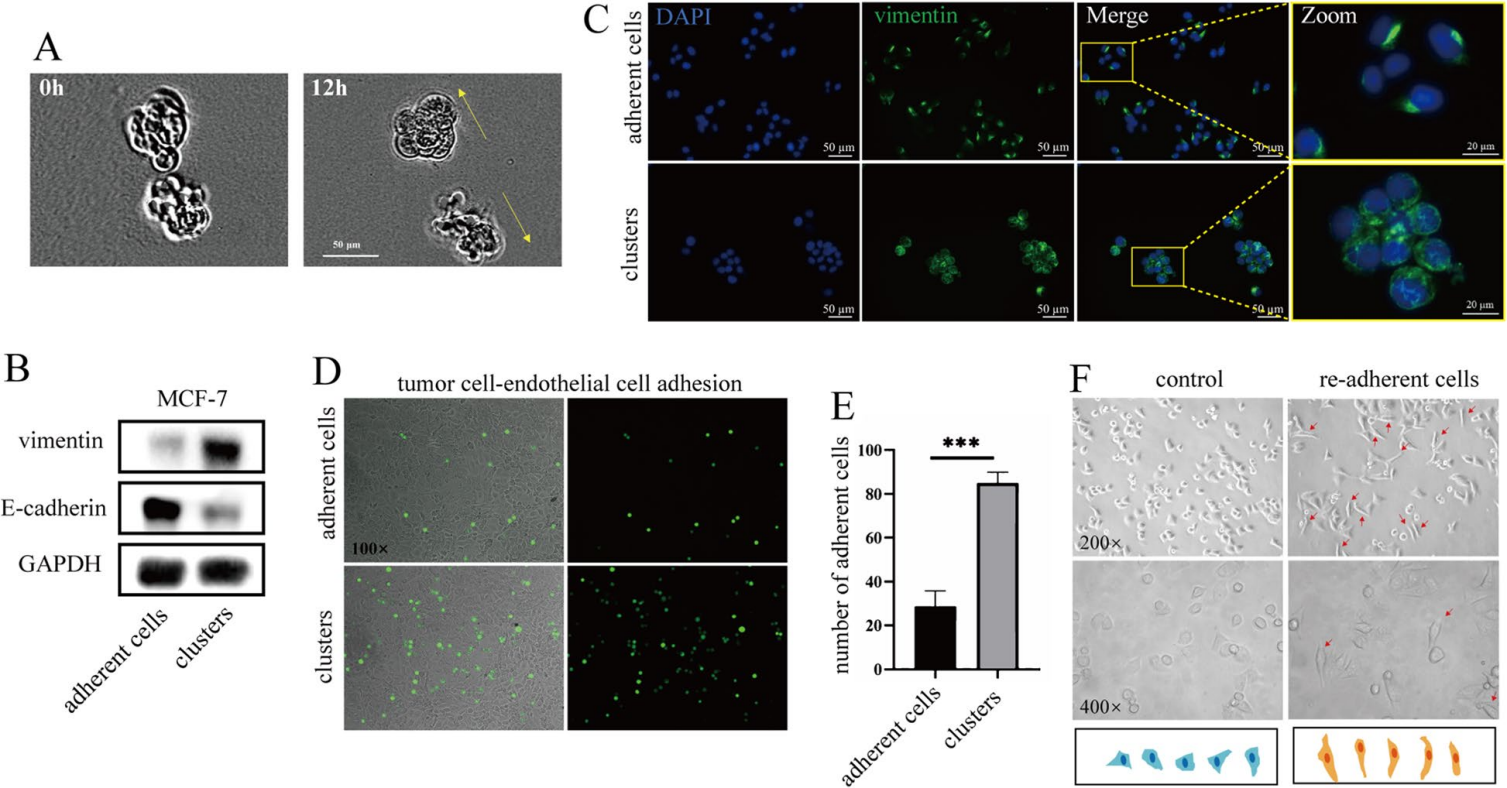
Evaluation of invasiveness of cell clusters in vitro. **A** Time-lapse images of cell clusters in 3D gels. The yellow arrow indicates the direction of movement of the cell clusters. **B** Western blot analysis of the protein levels of vimentin and E-cadherin in MCF-7 cells. **C** Immunofluorescence analysis of the vimentin localization in MCF-7 cells. DAPI (nuclei, blue), F-actin(green), Vimentin (green). **D**, **E** Tumor cell-endothelial cell adhesion test. The lower layer is HUVEC cells, and the upper layer is MCF-7-GFP cells (green). The quantitated results, *n* = 5, ***P < 0.001. Two tail student's t-test analysis was used to compare the statistical difference between indicated two groups. **F** Cell- reattachment assay. The red arrow point to the cells that become fusiform

We further found that cell clusters invaded as a whole by a gel invasion assay (Fig. 5A). One of the most striking features of metastasis was the transformation of cells from an epithelial phenotype to a mesenchymal phenotype by EMT. E-Cadherin as an epithelial marker was down-regulated while vimentin as a mesenchymal marker was up-regulated in cell clusters compared to adherent cells, suggesting the higher invasive capacity of the cell clusters compared to adherent cells (Fig. 5B & Figure S2C). Cellular immunofluorescence results also showed an up-regulation of vimentin expression and a wider range of expression sites (Fig. 5C). Notably, the up-regulation of vimentin in the cell clusters was not accompanied by a complete loss of expression of the epithelial marker E-Cadherin, suggesting that the cell clusters belonged to a more aggressive E/M hybrid phenotype.

In order to form distant metastases, CTCs must attach to secondary sites where they are retained by adhesion to endothelial cells, which is a prerequisite for CTC extravasation [32]. To assess the ability of cell clusters to extravasate out of the vasculature, the interaction of cell clusters with vascular endothelial cells was examined by a cell cluster-endothelial cell adhesion assay. A significant increase in the ability of cell clusters to adhere to endothelial cells was observed compared to apposed cells (Fig. 5D, E), indicating that cell clusters more readily adhered to vascular endothelial cells and extravasated out of the vasculature to distant organs. To examine the invasive ability of the cell clusters after transmigrating across the vessels, we took the cell clusters for re-adhesion experiments and cultured the cell clusters again in the apposed wall. The results indicated that the cell morphology changed from the original more round morphology to a shuttle shape (Fig. 5F), demonstrating that the invasive ability of the cells became stronger. The above results suggested that our cultured cell clusters had an up-regulated invasive capacity.

### Assessment of growth and proliferation capacity of CTC clusters in vitro

**Fig. 6:**
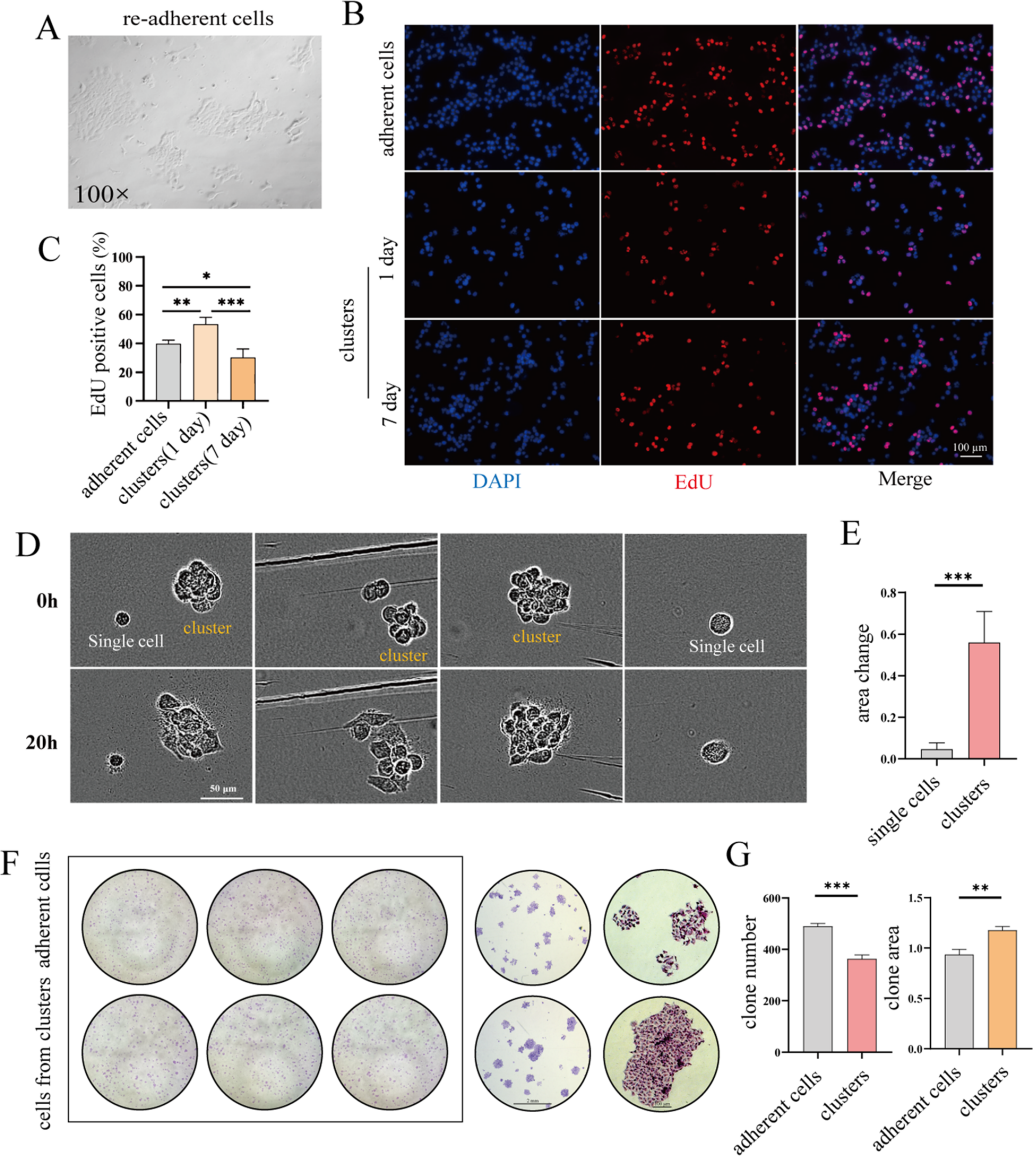
Evaluation of the growth and proliferation ability of cell clusters in vitro. **A** 4T1 cancer cells cultured in suspension for 5 days were switched back to the culture conditions of the adherent cells and their ability to grow again was observed. **B** Detection of proliferative cells in MCF-7 cell clusters by EdU cell proliferation assay. DAPI (nuclei, blue), EdU (red). The quantitated results are shown in (**C**), *n* = 5, *P < 0.05, **P < 0.01, ***P < 0.001. **D** Analysis of the growth ability of single cells and cell clusters in 3D gels by using time-lapse images. The quantitated results are shown in (**E**). *n* = 5, ***P < 0.001. **F** Colony formation of MCF-7 adherent cells and cells from MCF-7 cell clusters, the quantitated results are shown in (**G**), *n* = 3, **P < 0.01, ***P < 0.001. Two tail student's t-test analysis was used to compare the statistical difference between indicated two groups in (**E**) and (**G**), one-way ANOVA was applied in experiments containing multiple groups in (**C**)

The enhanced metastatic capacity of CTC clusters is ultimately reflected in the ability to colonize distant organs. In order to examine the ability of cell clusters to recolonize distant organs, cell clusters were subjected to re-adhesion experiments. As shown in Fig. 6A, the cell clusters had the ability to reattach and grow on the plates. Furthermore, as shown in Fig. 6B & C, the cell clusters remained proliferative and viable after 7 days of suspension by the visual EdU assay. The results were also consistent in 4T1 and MDA-MA-231 cells (Figure S2D, E, F). The three-dimensional (3D) gel experiments further demonstrated that the cell clusters had the ability to spread out and grow, while the single cell morphology did not change significantly (Fig. 6D, E) by the area change of the cells using time-lapse imaging. Next, we assessed the clonogenic ability of the cell clusters in vitro, and the experimental results showed that the cell clusters could form a similar number of clones as the adherent cells and the clone areas of cells from clusters were larger than that of single cells, indicating that the cells within the clusters had a greater tumorigenic capacity than single cells (Fig. 6F, G).

### Assessment of colonization capacity of CTC clusters in vivo

**Fig. 7:**
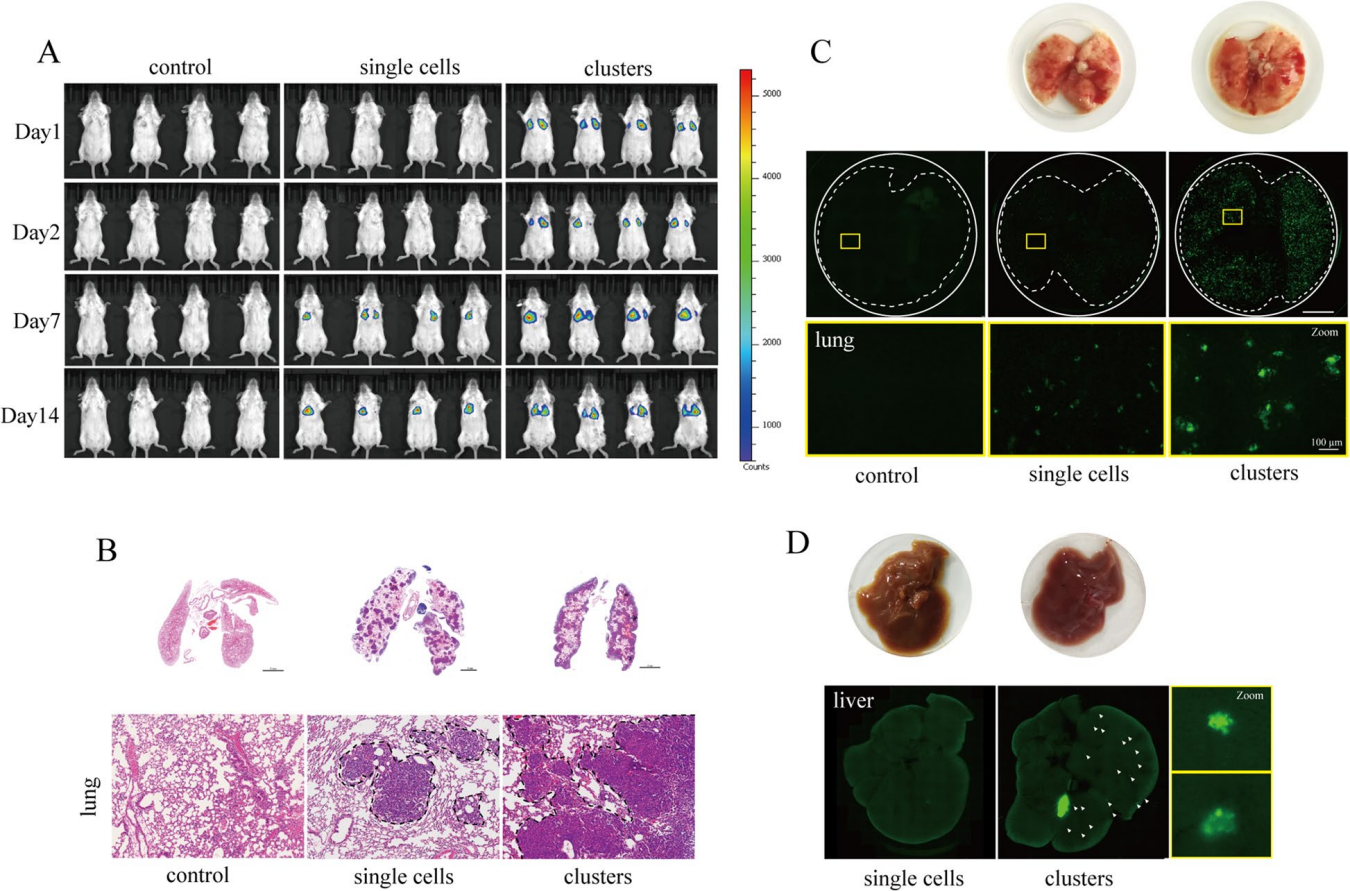
Evaluation of retention and colonization ability of cell clusters in vivo. **A** Representative bioluminescence images of mice at different time points after tail vein injection with luciferase-expressing 4T1 cells. **B** Representative images of H&E-stained sections of mouse lungs in groups injected with single cells or cell clusters, bottom: individual metastasis areas. **C** Up: lungs 3 days after tail vein injection of 4T1-GFP single cells or cell clusters, down: individual metastasis areas. 4T1-GFP cell clusters arrested in the lung (green). **D** Up: Livers 3 days after tail vein injection of 4T1-GFP single cells or cell clusters, the white arrows point to 4T1-GFP cell clusters arrested in the Liver. down: individual metastasis areas

We also examined whether these metastatic properties of cell clusters were consistent with in vivo using the experimental metastasis model. As shown in Fig. 7A, the fluorescence density in the lung of mice with the injection of cell clusters was significantly higher than that in the lung of mice with the injection of single cells, and the fluorescence was detected on day 1 in mice with cell clusters, while did until day 7 in the group with single cells. This result suggested that the cell clusters were more easily intercepted by the capillaries and colonized in the lungs. The lungs of mice were taken for pathological staining and the results were consistent with live imaging (Fig. 7B). It is concluded that the cancer cell clusters have a stronger colonization capacity than single cells. To visualize the retention of cells in the lungs, we injected 4T1-GFP cells into the tail vein of the mice and collected the lungs after 3 days for ex vivo fluorescence imaging. The image data showed that more cells with GFP were detected in the lungs of the mice injected with cell clusters rather than with individual cells (Fig. 7C). Several metastatic foci were also present in the liver of mice injected with the cell cluster group, whereas none in the single cell group (Fig. 7D). The above results indicated that the cell cluster model we constructed in vitro had the same enhanced metastatic colonization capacity as spontaneous CTC clusters in vivo.

## Discussion

Since previous methods for culturing cell clusters in vitro have not comprehensively considered the influence of the environment on cell phenotypes, here we have enriched the culture conditions based on the physiological environment of CTCs in the circulation. We used the similarity of the environment to induce in vitro CTC cluster models to produce phenotypes similar to those of clinical CTCs, constructed a more scientific and economical method for cluster culture, and proposed for the first time to reveal the molecular characteristics of CTC clusters as a reference. The requirement to assess the similarity of molecular features and cellular behaviors between cell clusters and clinical CTC clusters in various stages of metastasis helped the researchers to ensure that the cell cluster can truly reflect the unexplored molecular features of CTC clusters when using the cell cluster model for in-depth molecular characterization. Although the feasibility of an in vitro replacement model of CTC clusters was previously noted in the literature [38], only morphological similarities were briefly analyzed, without taking into account the cellular behavior of CTC clusters during metastasis, and the important and complex roles of adhesion proteins.

CTC cell lines have unique intermediate properties between primary tumor and distant metastases, and it is difficult to deny the important role of CTC cell lines in the study of CTC biology. However, because CTC isolation techniques and in vitro culture remain challenging, only a limited number of CTC permanent cell lines are currently available and are much more difficult to manage than standard cell lines [9]. Notably, because optimal culture conditions for CTCs have not been determined, current studies rely on stem cell culture methods to ensure maximum CTC expansion. Each culture protocol has been designed based on the researchers' own expertise and empirical results, rather than on the theoretical basis of the biological properties of CTCs, and the CTC cell line culture conditions still need to be further optimized.

It is urgent to design therapeutic strategies to reduce the metastatic capacity of CTC clusters by targeting their molecular characteristics. Therefore, there is requirements to promote the development of in vitro simulation CTC cluster culture methods. Cancer cell lines grown in suspension as cell clusters can facilitate the development of such approaches because the cells in these clusters exhibit the same adhesion mechanisms as those in CTC clusters, and based on these similarities, we believed that established cell lines grown in suspension as cell clusters can offer a range of advantages that have not yet been explored. Because they are easy to culture and analyze, many general aspects related to clinical CTC clusters can be studied in these cell lines. Already, a number of researchers have used cell line generated cell cluster models to study potential therapeutic targets of CTC clusters and to validate the effect of drugs on CTC cluster dissociation [39]. This alternative approach is able to help researchers break the limitations and freely study the molecular properties of CTC clusters and find more agents to reduce hematogenous metastasis.

When culturing cell clusters in vitro, the growth environment of cells becomes a key factor in the ability to induce a similar cell phenotype. We first considered the nutritional conditions for cell growth, we combined a low concentration of fetal bovine serum with a low concentration of growth factors to make a richer nutritional source and to fit the physiological conditions. Shaking is also a key condition to simulate the intravascular fluid environment. Fluid shear stress provided by fluid shaking is an important factor in inducing phenotypic changes in CTC clusters in the blood [37], in addition, cancer cells tend to loosely bound into clusters and easily disperse without shaking, making them unsuitable for further studies. In addition, hypoxia is a major characteristic of the blood environment. Hypoxia can activate the EMT signaling pathway, stimulate the expression of adhesion proteins, and promote metastasis. Hypoxia exists within CTC clusters and hypoxic CTC clusters are more likely to invade and extravasate from the vasculature [31]. However, the researchers only added hypoxic conditions to the culture of CTC permanent cell lines, and the majority of in vitro CTC cluster models were still cultured using normoxic conditions, by which failed to induce an effective cell phenotype.

Admittedly, there are limitations to the use of in vitro CTC cluster models. The in vitro CTC cluster models do not provide insight into the first phase of the metastatic process (collective shedding of tumor cells). However, they can help us understand the more important stage of "colonization". For example, researchers have used them to reveal the mechanism by which cells in CTC clusters cooperate with each other to promote growth capacity [40], providing strong evidence for the high metastatic capacity of CTC clusters. It has to be admitted that this manuscript does not aim to capture CTC clusters in patients or animals for rigorous molecular characterization in comparison with in vitro CTC cluster models, the main obstacle being the scarcity of CTC clusters in patients or animals, which makes it more difficult to capture sufficient clusters of cells for experiments. We also tried to capture spontaneous CTC clusters in hormonal mice, but the first time a similar CTC cluster is captured, it has to be identified by immunofluorescence experiments, and only pan-CK^+^ and CD45^−^ cells are CTCs, and this identification step affects the subsequent experiments. Certainly, the comparative metrics addressed in this manuscript are reported in many literature analyzing the molecular characterization of CTC clusters with abundant supporting evidence, and therefore, we chose to build on the existing literatures by comparing the many biological features without performing such a difficult and non-essential task as capturing the CTC clusters in the patient's body. However, when using cell clusters generated from cancer cell lines as an initial exploratory model, subsequent experiments using animal and human primary tumor cells for validation are necessary, and only in this way can the role of the in vitro model be implemented into the clinic to uncover new targets of clinical significance.

Some individual studies have added neutrophils to the in vitro CTC cluster model to construct a CTC-neutrophil model [39], but there is the same problem with the scientific nature of the cultivation conditions. Most studies have constructed in vitro models of CTC clusters without considering the presence of non-cancer cells, such as, platelets [19], fibroblasts [41], and immune cells [42], and the presence of these cells is thought to affect the nature and therapeutic response of true CTC clusters. It has been shown that these non-cancer cells can help CTCs to metastasize. To better understand the metastasis-promoting role of these non-cancerous cells, additional and scientific heterotypic cluster culture methodologies for revealing the effect of non-cancerous cells on CTCs need to be developed in the future. Finally, the use of different cell sources to generate in vitro CTC cluster models may also lead to different findings. We need to validate these new findings on different cellular models, as well as explore their clinical value in the context of clinical data.

Although disintegration of CTC clusters into individual cells can reduce their metastatic potential, this approach is still modest because individual CTCs still have the potential to form metastases. We hope to use these in vitro CTC cluster models to investigate more effective ways for targeting CTC clusters. To achieve this goal, we need to further characterize the adhesion molecules that lead to CTC cluster formation, as well as the genetic and biological characteristics of the tumor cells that carry these molecules.

CTC clusters have strong dynamic changes in their molecular characteristics during metastasis in order to adapt to different microenvironment, and the dynamic changes of their adhesion proteins at different times are well worth in-depth studies. Considering the adherent cells as tightly packed in situ tumor and the cells in suspension culture as CTCs, the altered molecules can be detected and the dynamic changes can be better observed by changing the culture conditions. In addition, the biological details of CTC cluster generation, propagation in circulation, and metastasis formation in distant organs need to be further investigated.

## Conclusions

We optimized the culture conditions of in vitro CTC clusters model and proposed a method for evaluating in vitro CTC clusters based on the reported biological characteristics of clinical CTC clusters. The in vitro CTC clusters model constructed using breast cancer cell lines was found to have molecular features partially consistent with those of reported CTC clusters when compared with individual cells, and this evaluation of the model could improve the reliability of in vitro CTC clusters.

### Supplementary Information

**Additional file 1: Figure S1.** The molecular characteristics of CTC clusters in MDA-MB-231 and 4T1 cells. (**A**) Representative images of the appearance of 4T1 cell clusters and CTC clusters from patients. F-actin (green), DAPI (nuclei, blue). **B **TEM images of 4T1 cell clusters. J, cell-cell junction. The red arrow points to the tight junction. **C **Western blot analysis of the protein levels of plakoglobin in MDA-MB-231 and 4T1 adherent cells and cell clusters. Immunofluorescence analysis of the protein levels difference of plakoglobin (**D**) and ZO-1 (**E**) in MDA-MB-231 and 4T1 adherent cells and cell clusters. DAPI (nuclei, blue), plakoglobin (red), ZO-1 (red).

**Additional file 2: Figure S2.** The related proteins expression and proliferative characters of CTC Clusters. **A **Immunofluorescence analysis of the protein levels of PD-L1 in MCF-7 cells. DAPI (nuclei, blue), PD-L1(green). **B **The chip captures MCF-7-GFP cell clusters. Scale bars, 20/50μm. GFP (green), DAPI (nuclei, blue). **C** Western blot analysis of the protein levels of E-cadherin in 4T1 cells. Detection of proliferative cells in 4T1 (**D**) and MDA-MB-231 (**E**) cell clusters by EdU cell proliferation assay. DAPI (nuclei, blue), EdU (red). The quantitated results are shown in (**F**), n = 5, **P < 0.01. Two tail student's t-test analysis was used to compare the statistical difference between indicated two groups.

**Additional file 3: Figure S3.** Representative images of Circulating Tumor Cells (CTCs) clusters captured in breast cancer patients. Immunofluorescence staining detects the expression of Pan-CK and CD45 in cells, with Pan-CK+ and CD45- indicating circulating tumor cells (CTCs), and Pan-CKand CD45+ indicating white blood cells (WBCs). The image shows clusters of CTC-WBC.

**Additional file 4.**

## Data Availability

The datasets used during the current study are available from the corresponding author on reasonable request.

## Abbreviations

CTC: Circulating tumor cell
ULA: Ultralow-attach
E/M hybrid phenotype: Epithelial/mesenchymal hybrid phenotype
PG: Plakoglobin
CEACAM6: Carcinoembryonic antigen related cell adhesion molecule 6
PD-L1: Programmed cell death-ligand 1
LDA: A limiting dilution assay
DIC: Differential Interference contrast
EdU: 5-Ethynyl-20-deoxyuridine
EGF: Epidermal growth factor
bFGF: Basic fibroblast growth factor
TEM: Transmission electron microscopy
HIF-1α: Hypoxia-inducible factor-1α
EMT: Epithelial-mesenchymal transition
3D: Three-dimensional
CSCs: Cancer stem cells
Pan-CK: Pan-Cytokeratin
