# Supplementary figures and images for: Systematic optimization and evaluation of culture conditions for the construction of circulating tumor cell clusters using breast cancer cell lines
Source: BMC Cancer. 2024 Apr 23;24:507. doi: 10.1186/s12885-024-12214-9 (PMC11036701; doi:10.1186/s12885-024-12214-9)

A

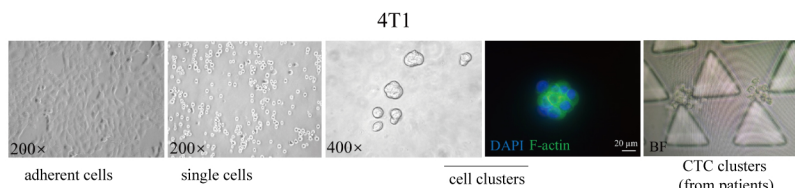

B

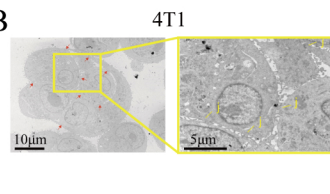

C

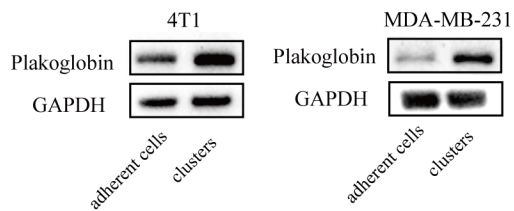

D

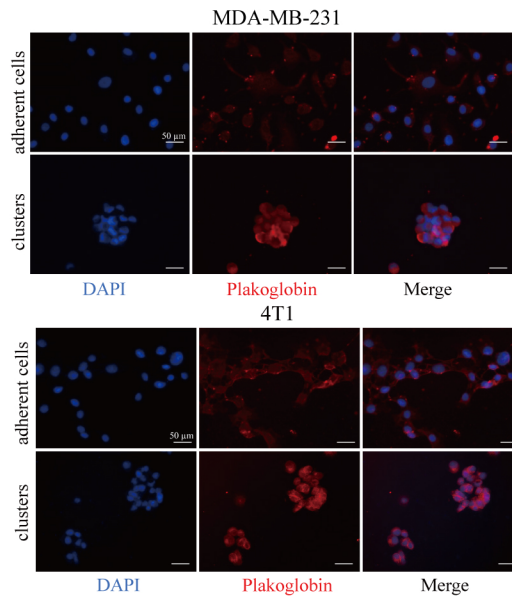

E

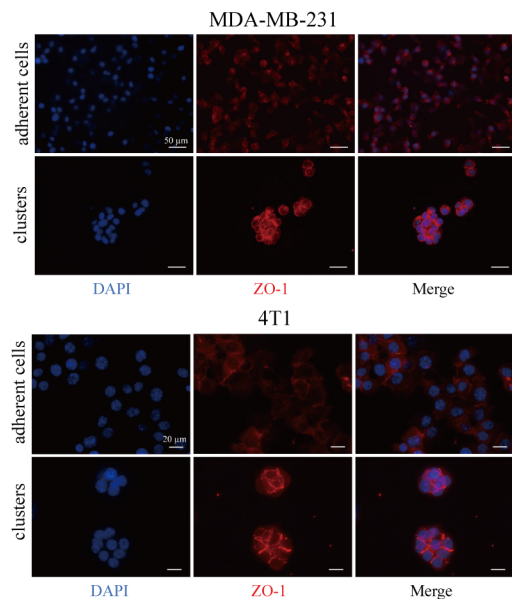

Supplement: Supplementary file 1 — Additional file 1: Figure S1. The molecular characteristics of CTC clusters in MDA-MB-231 and 4T1 cells. (A) Representative images of the appearance of 4T1 cell clusters and CTC clusters from patients. F-actin (green), DAPI (nuclei, blue). B TEM images of 4T1 cell clusters. J, cell-cell junction. The red arrow points to the tight junction. C Western blot analysis of the protein levels of plakoglobin in MDA-MB-231 and 4T1 adherent cells and cell clusters. Immunofluorescence analysis of the protein levels difference of plakoglobin (D) and ZO-1 (E) in MDA-MB-231 and 4T1 adherent cells and cell clusters. DAPI (nuclei, blue), plakoglobin (red), ZO-1 (red). [file 12885_2024_12214_MOESM1_ESM.pdf]

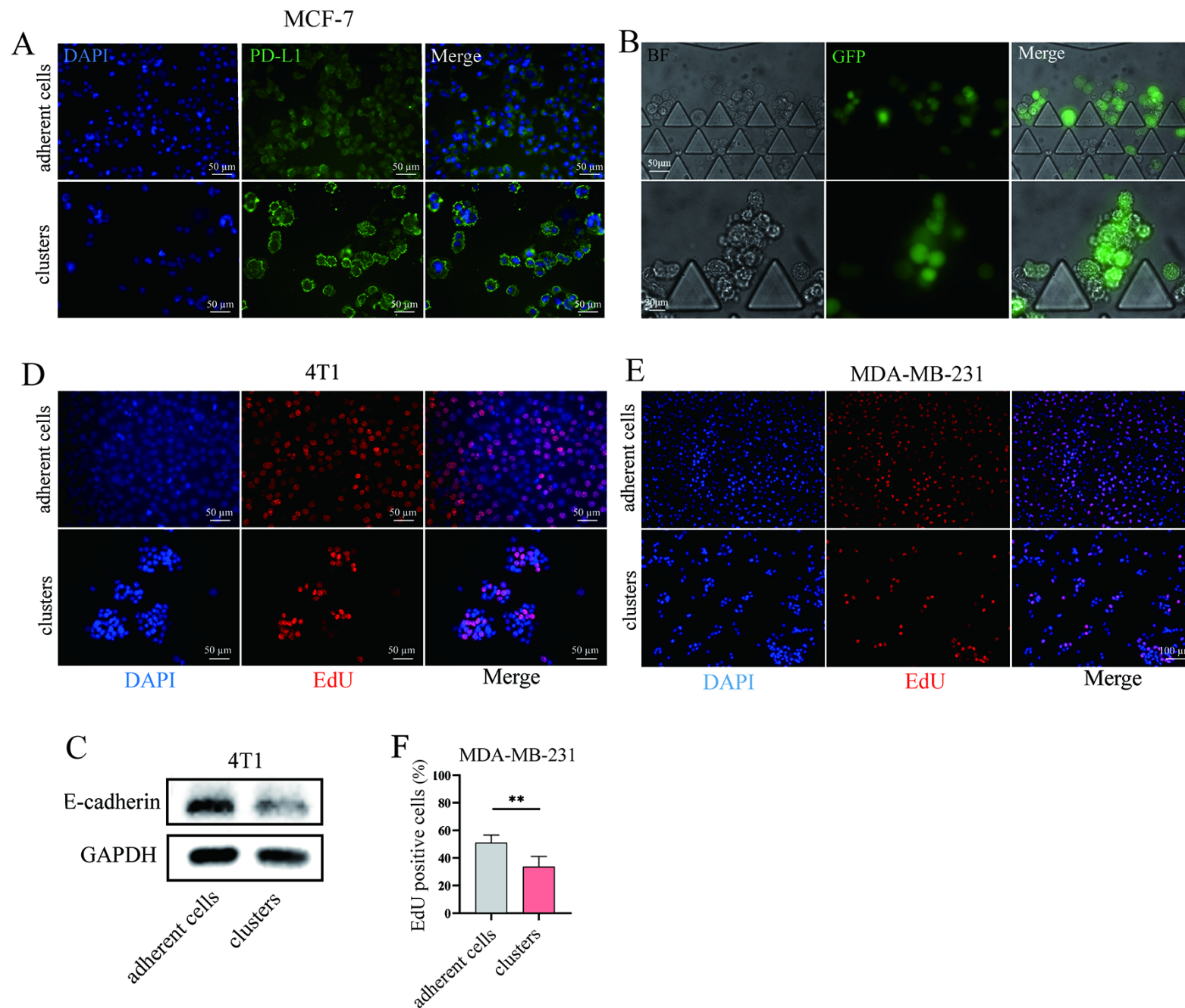

Supplement: Supplementary file 2 — Additional file 2: Figure S2. The related proteins expression and proliferative characters of CTC Clusters. A Immunofluorescence analysis of the protein levels of PD-L1 in MCF-7 cells. DAPI (nuclei, blue), PD-L1(green). B The chip captures MCF-7-GFP cell clusters. Scale bars, 20/50μm. GFP (green), DAPI (nuclei, blue). C Western blot analysis of the protein levels of E-cadherin in 4T1 cells. Detection of proliferative cells in 4T1 (D) and MDA-MB-231 (E) cell clusters by EdU cell proliferation assay. DAPI (nuclei, blue), EdU (red). The quantitated results are shown in (F), n = 5, **P < 0.01. Two tail student's t-test analysis was used to compare the statistical difference between indicated two groups. [file 12885_2024_12214_MOESM2_ESM.pdf]

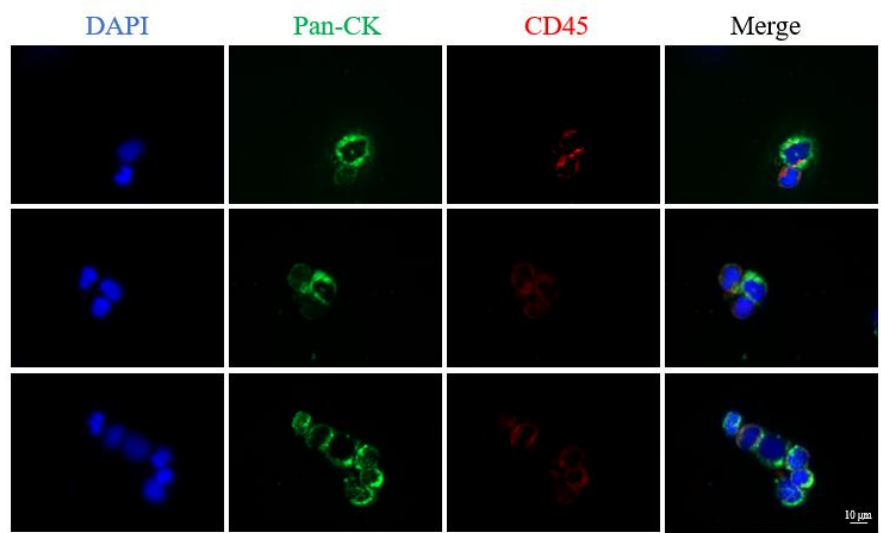

Supplement: Supplementary file 3 — Additional file 3: Figure S3. Representative images of Circulating Tumor Cells (CTCs) clusters captured in breast cancer patients. Immunofluorescence staining detects the expression of Pan-CK and CD45 in cells, with Pan-CK+ and CD45- indicating circulating tumor cells (CTCs), and Pan-CKand CD45+ indicating white blood cells (WBCs). The image shows clusters of CTC-WBC. [file 12885_2024_12214_MOESM3_ESM.pdf]

figure 1E

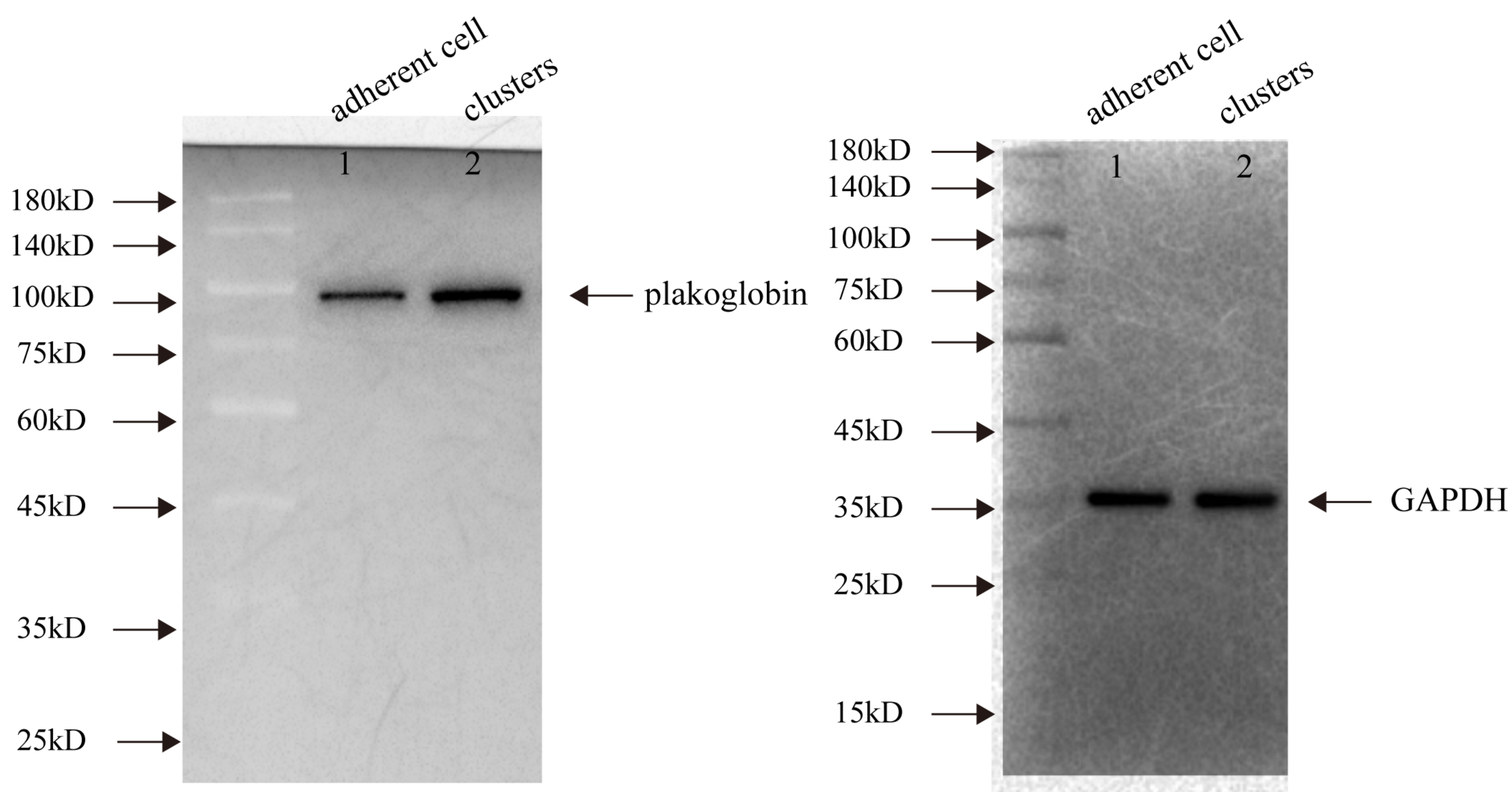

figure 2A

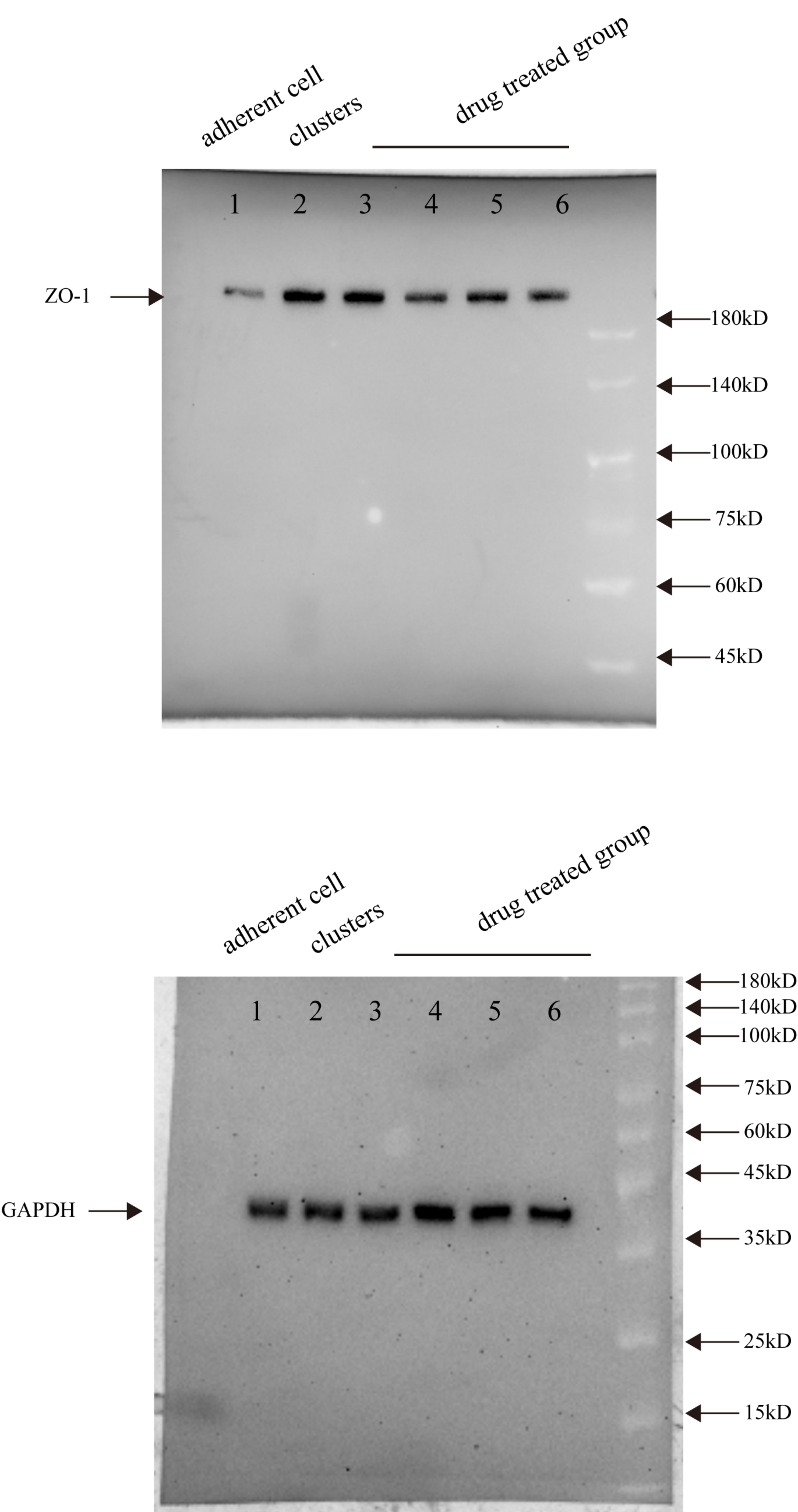

figure 2A

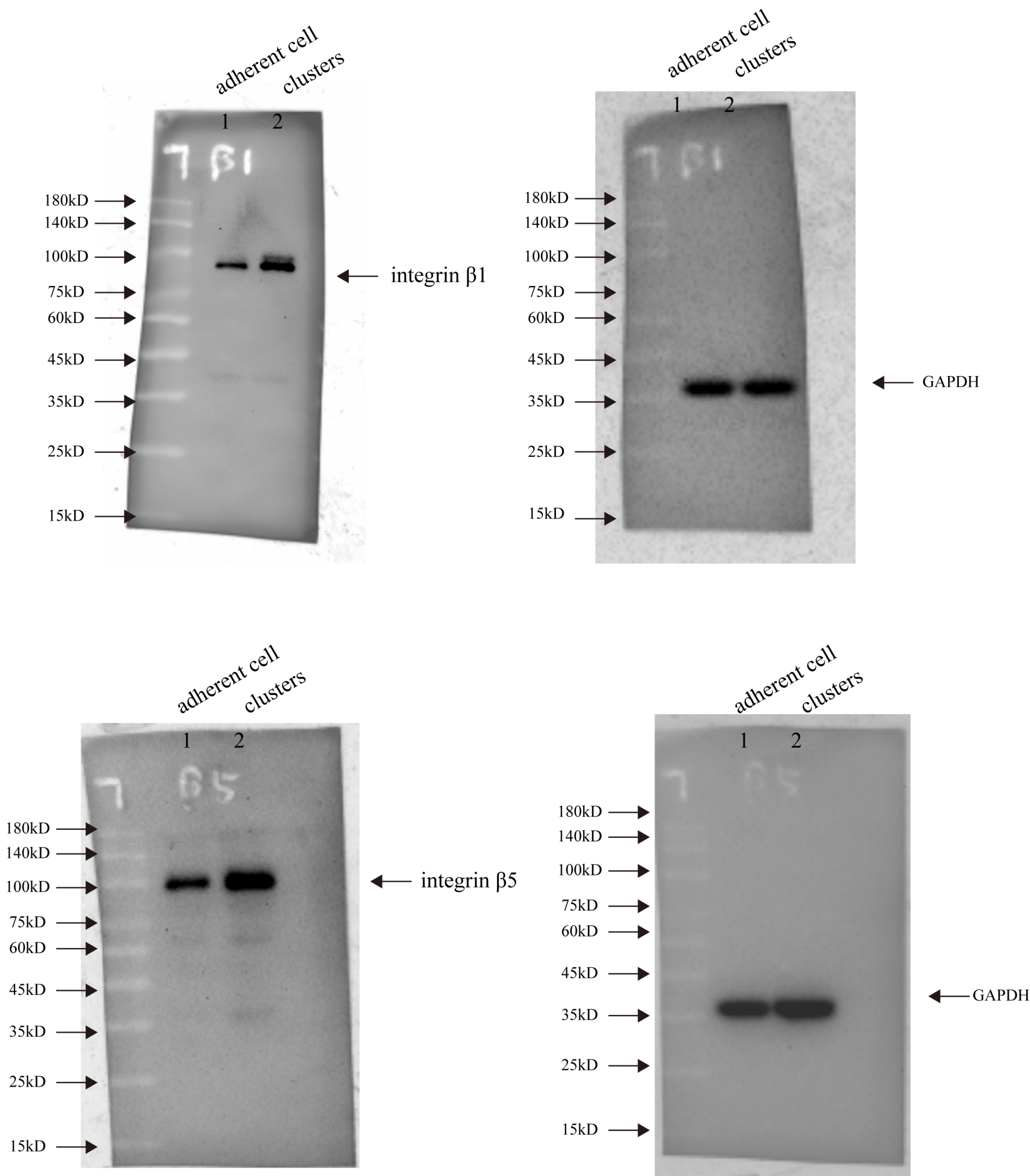

figure 2A

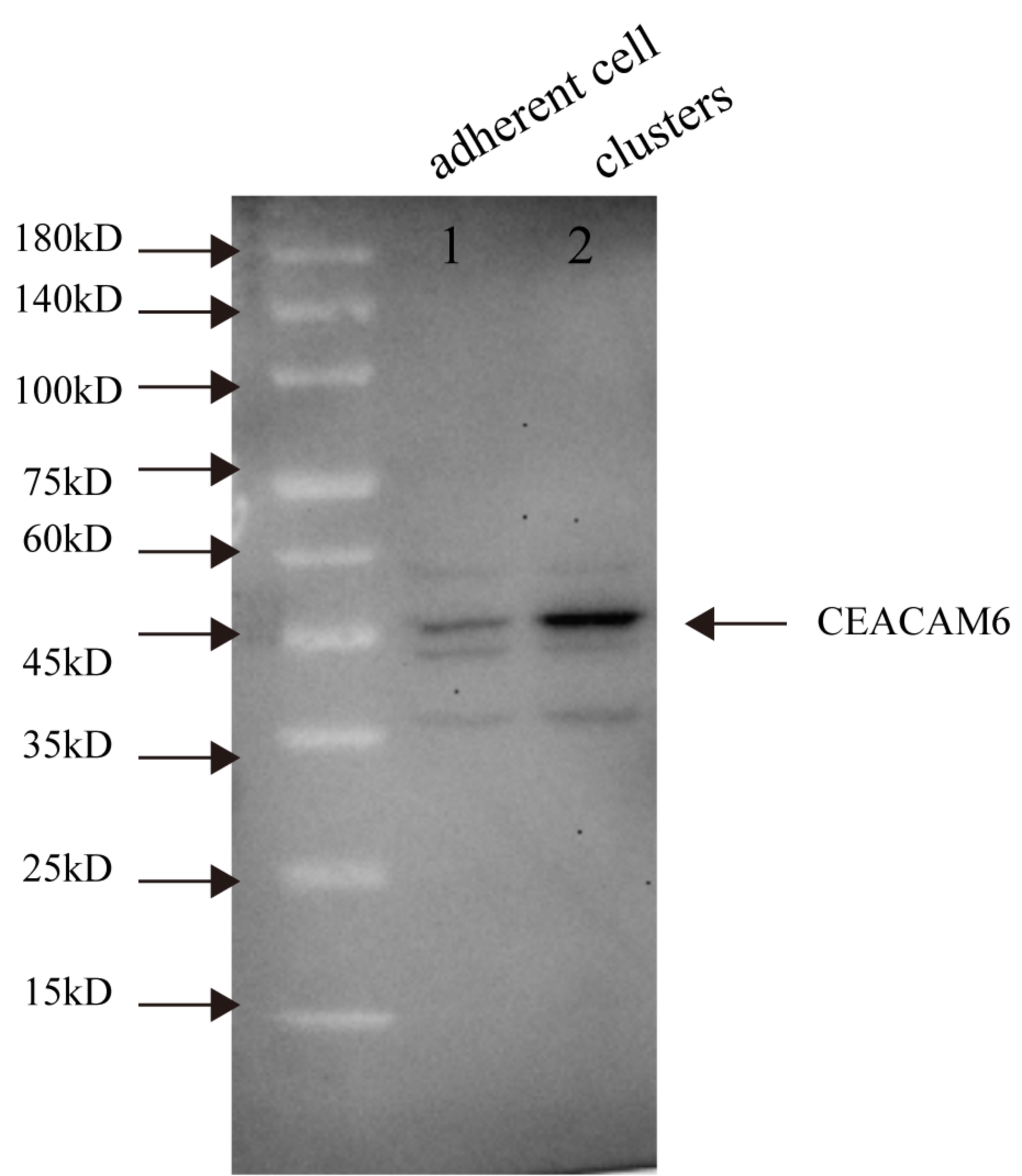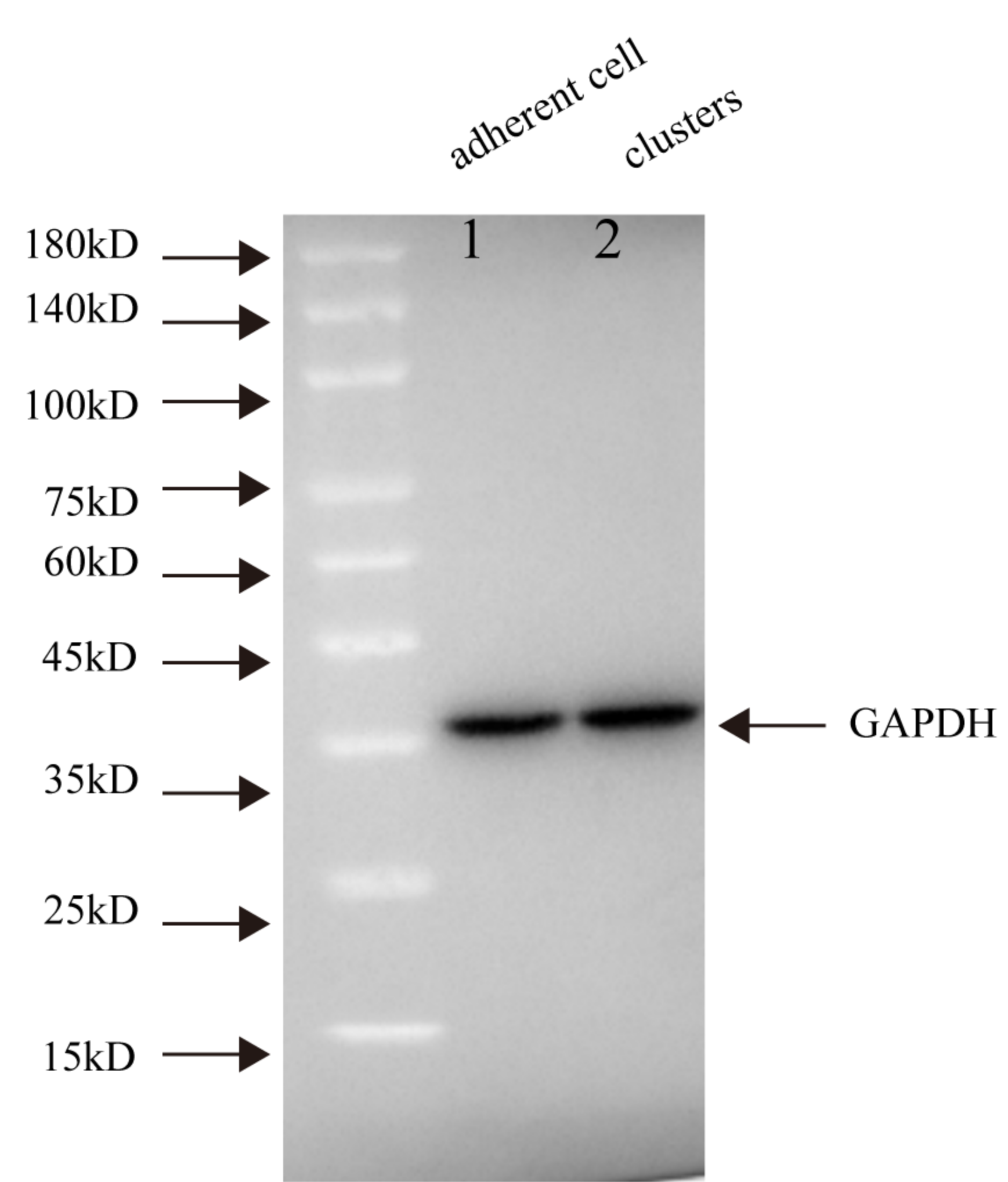

figure 3G

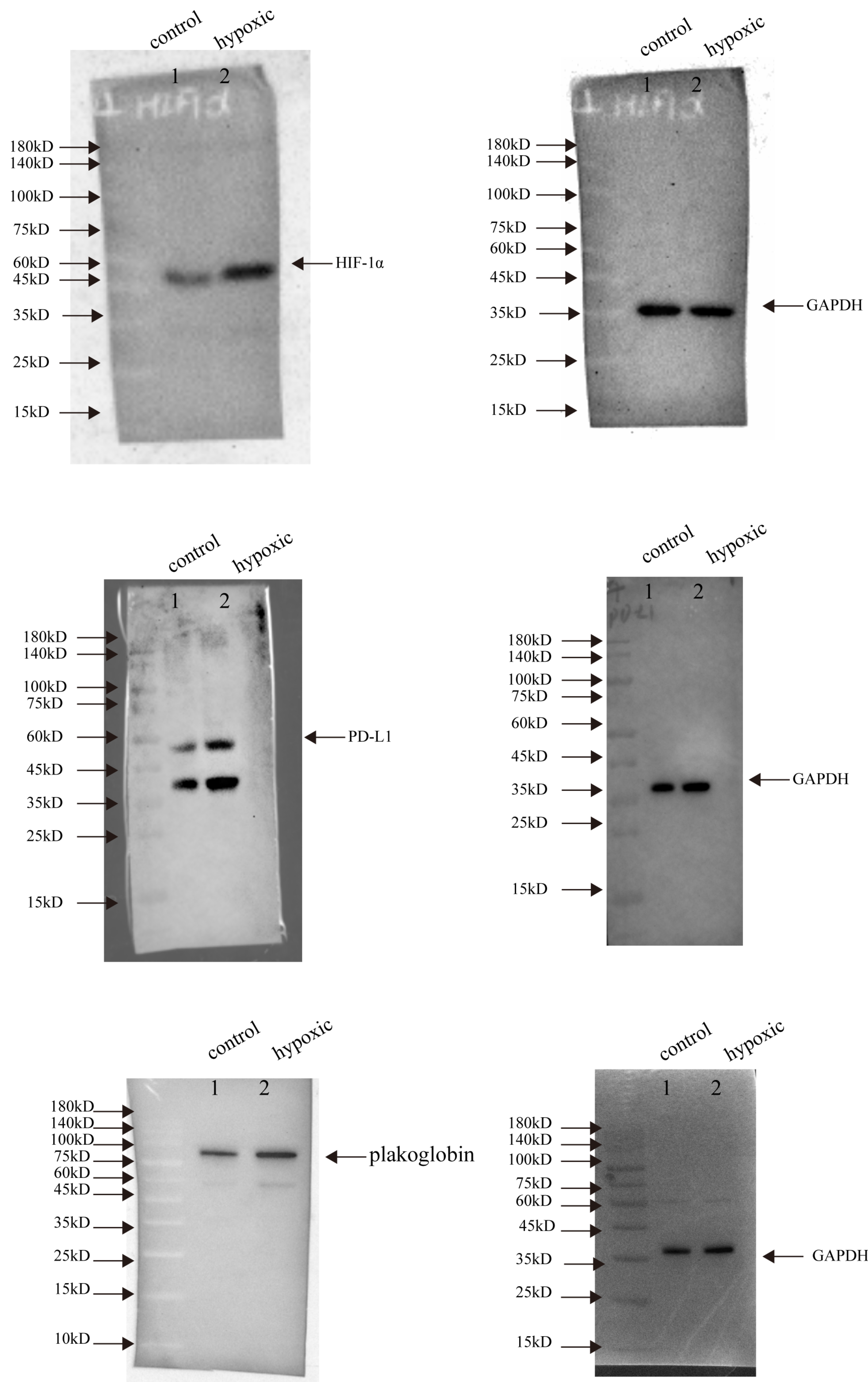

figure 4B

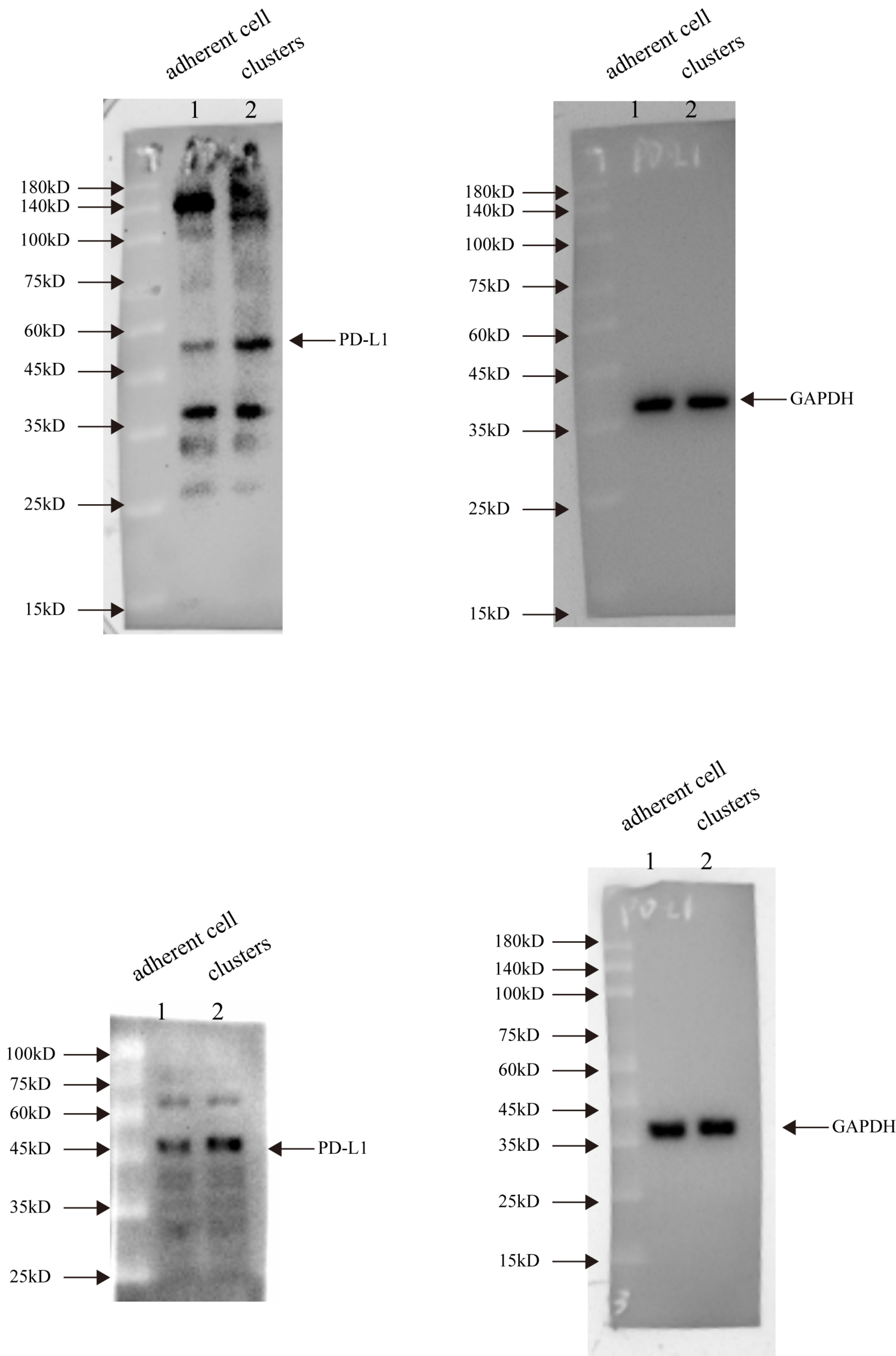

figure 5B

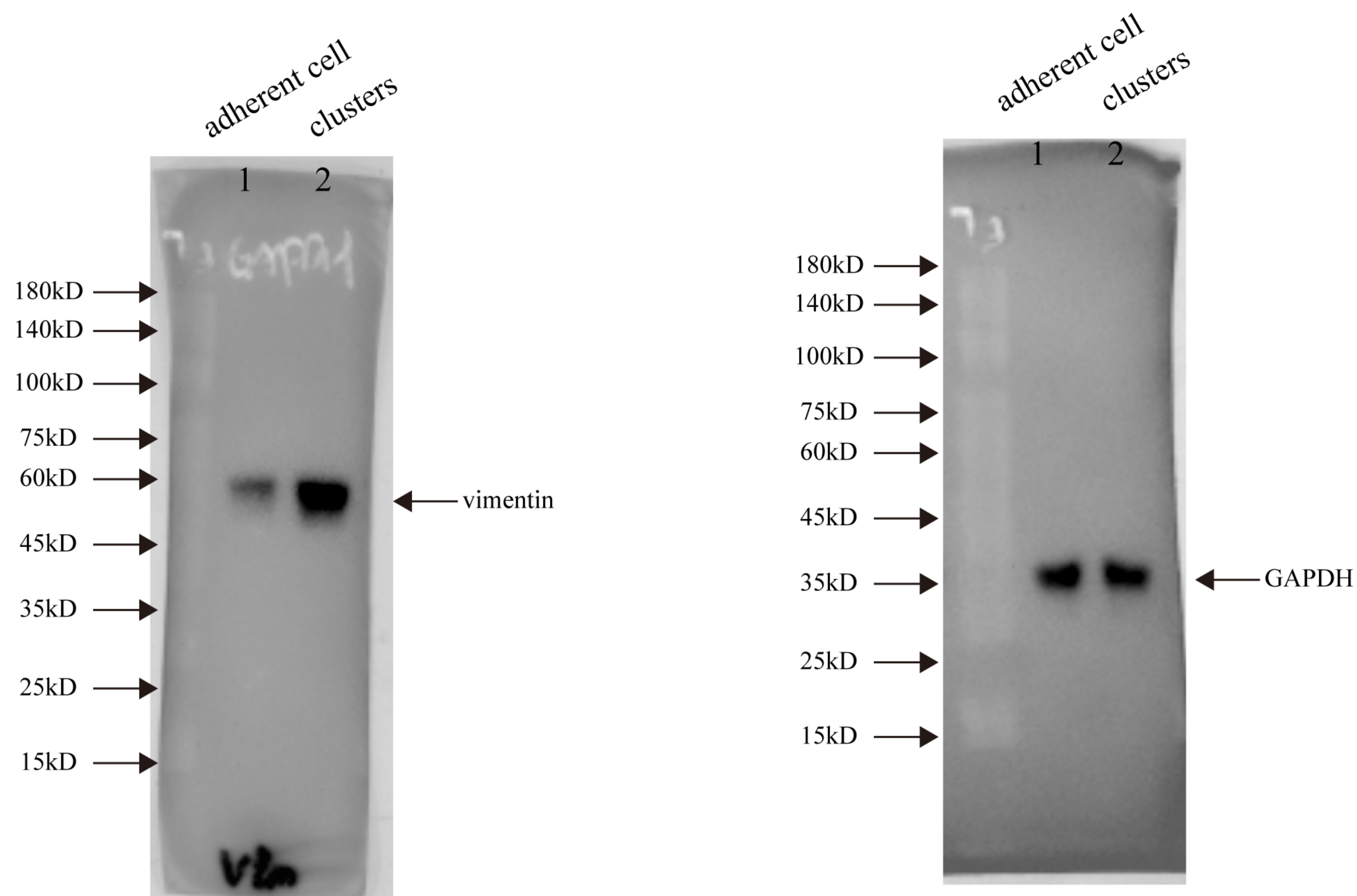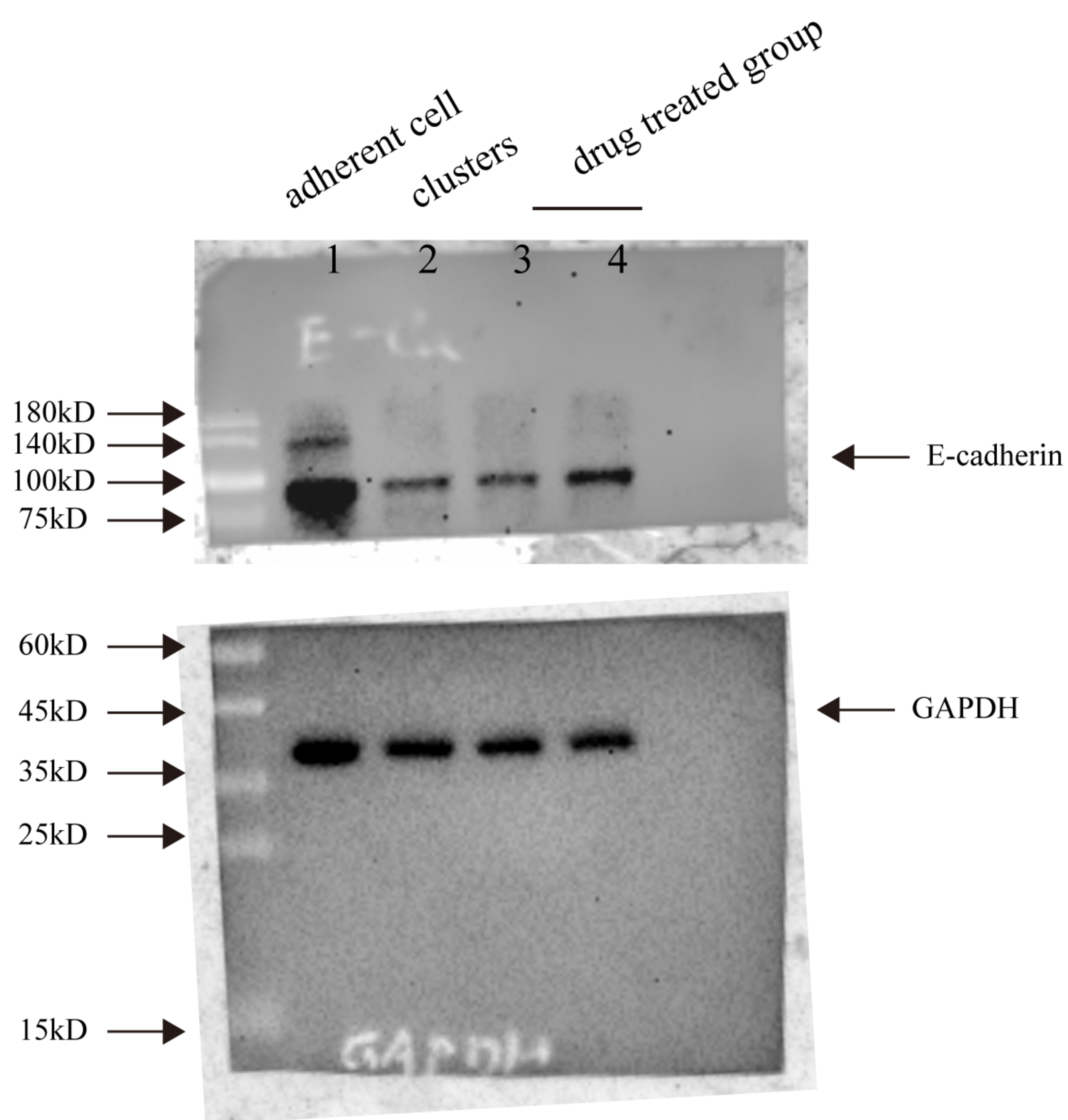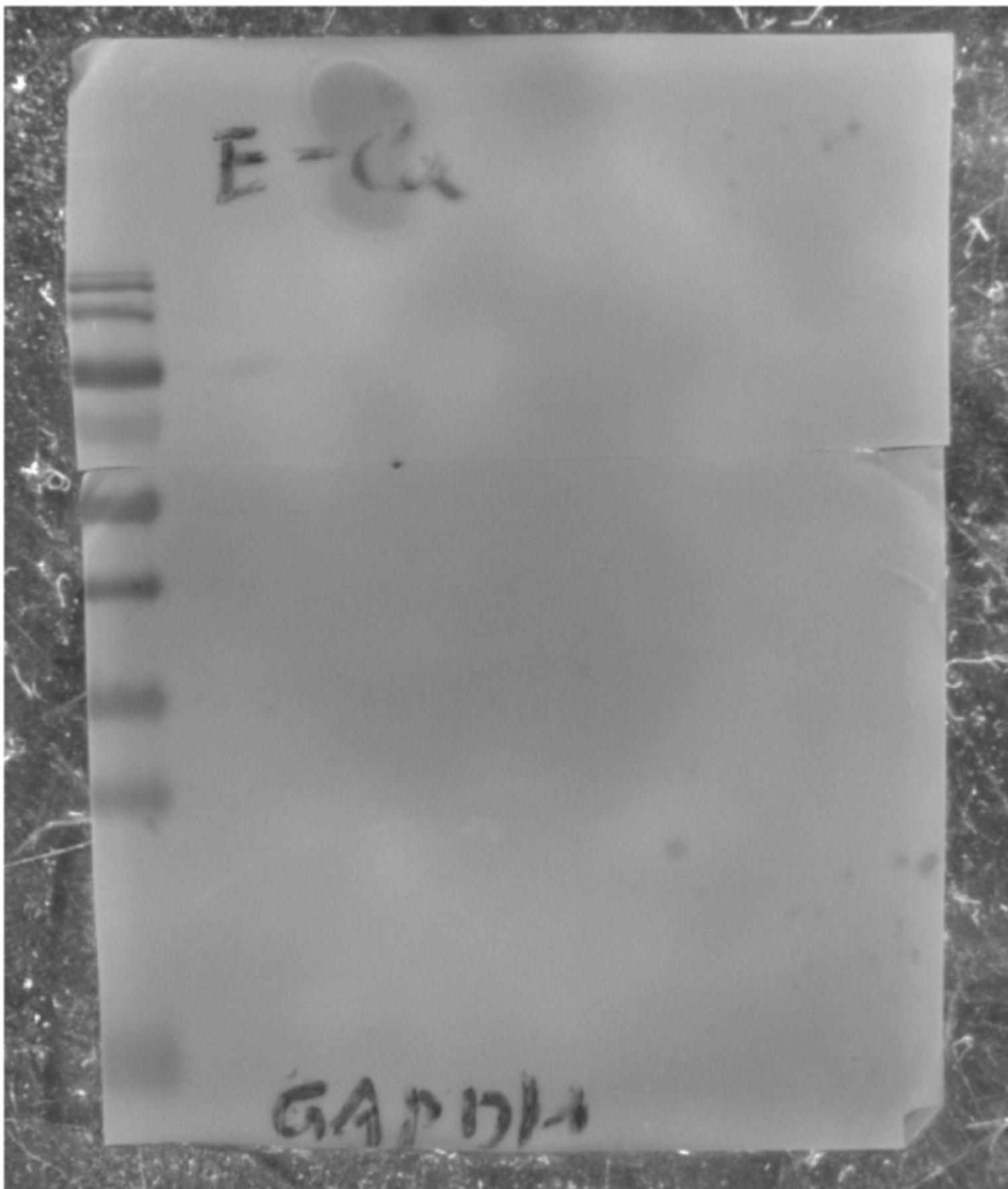

figure S1

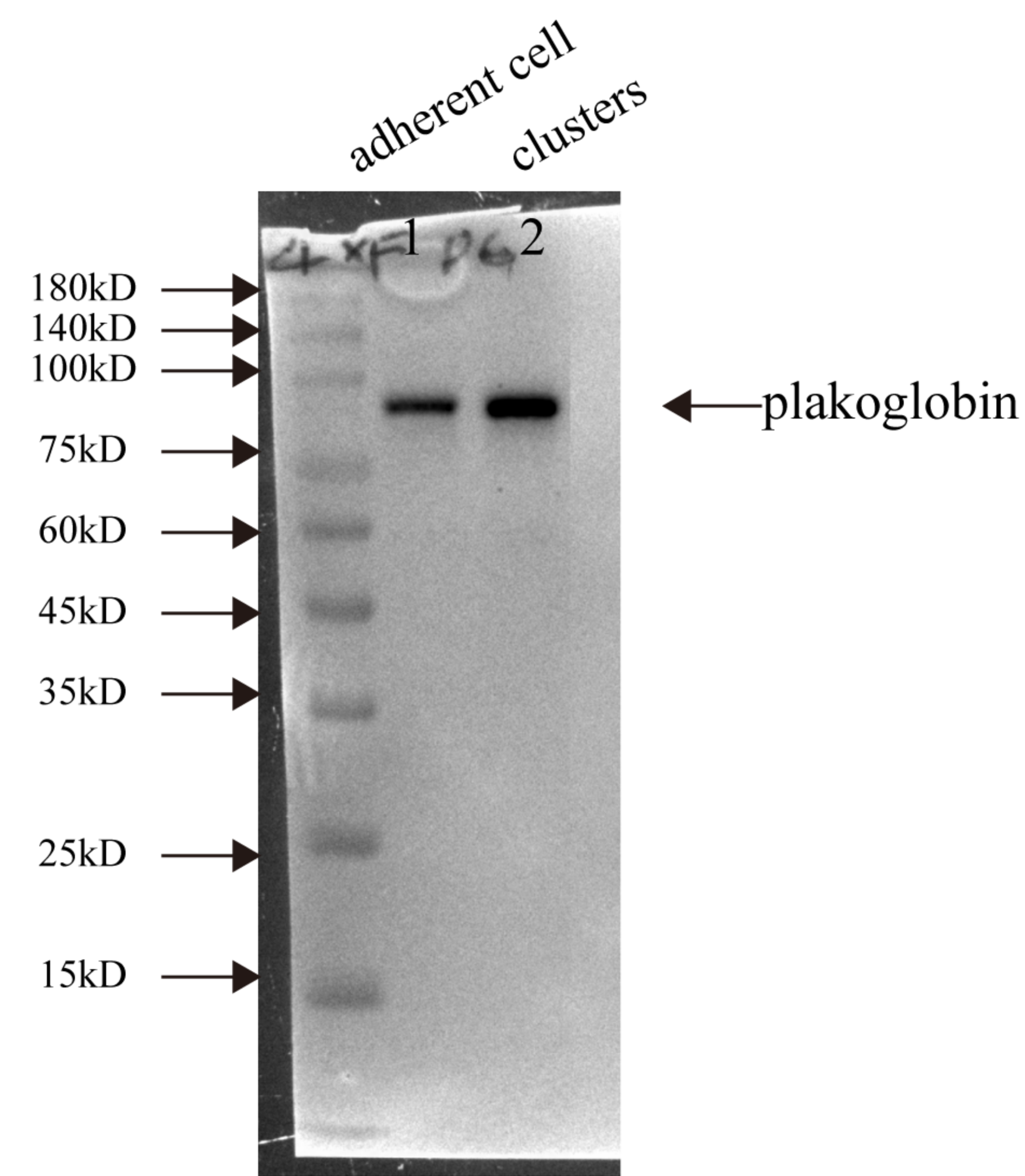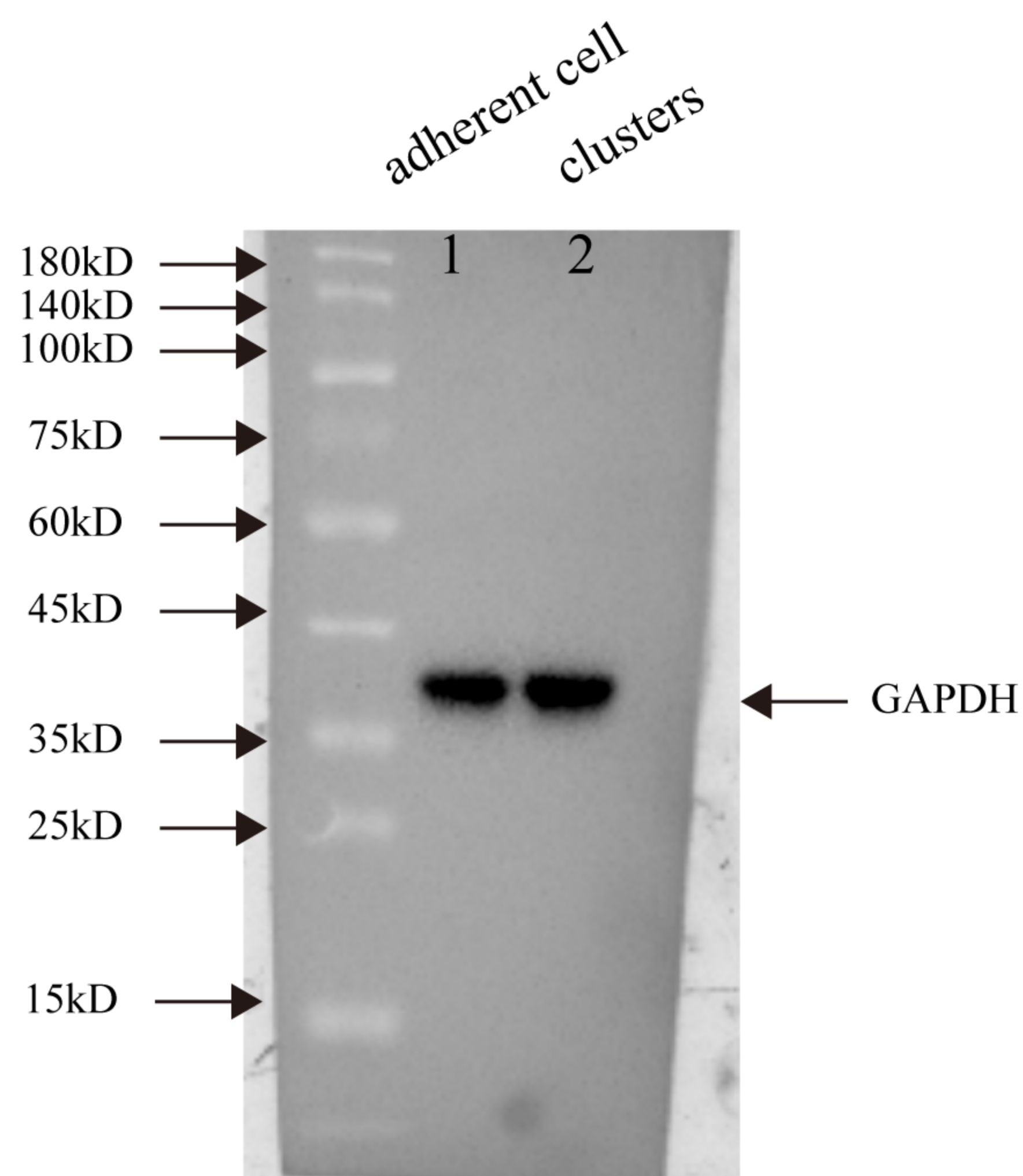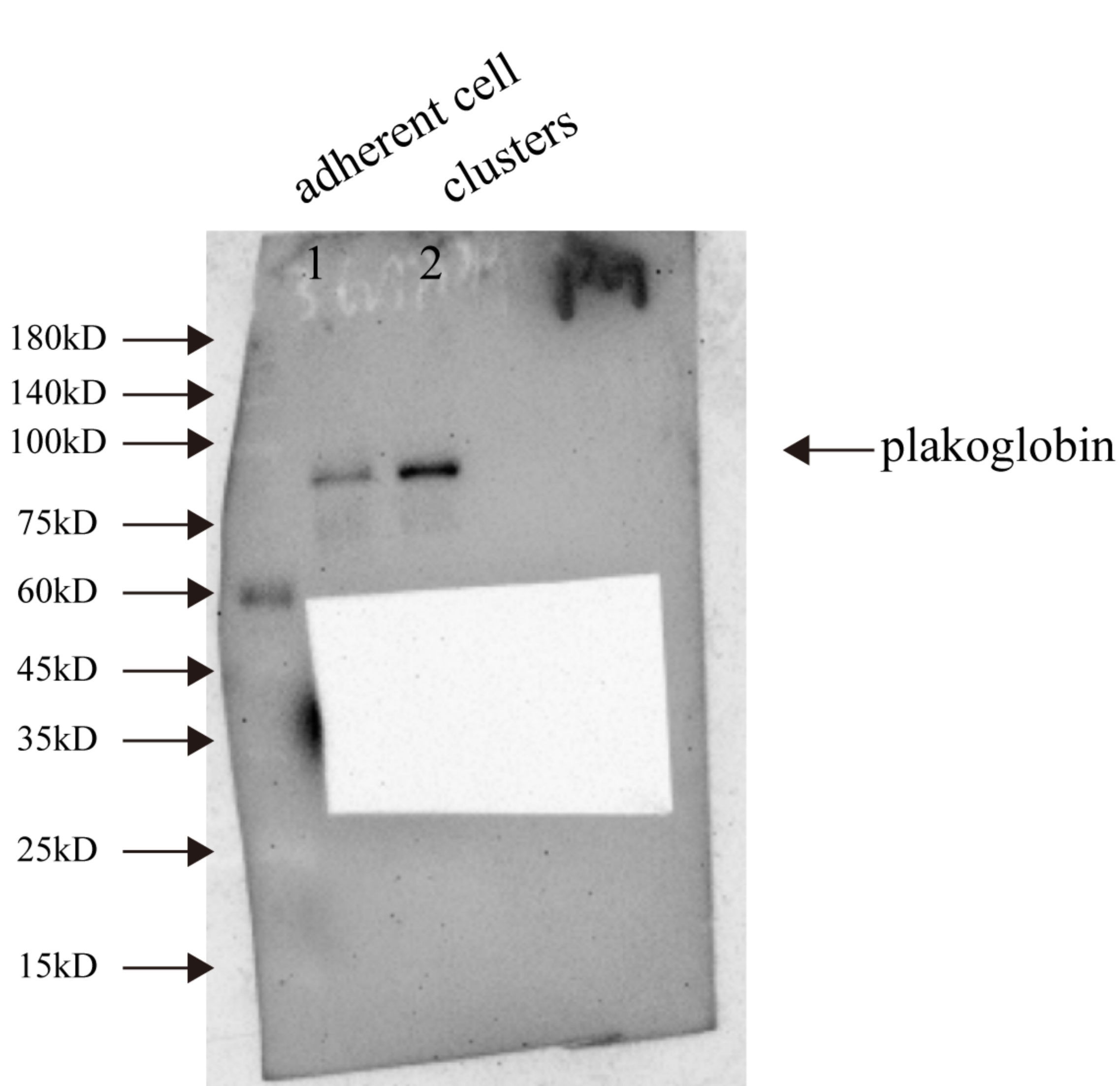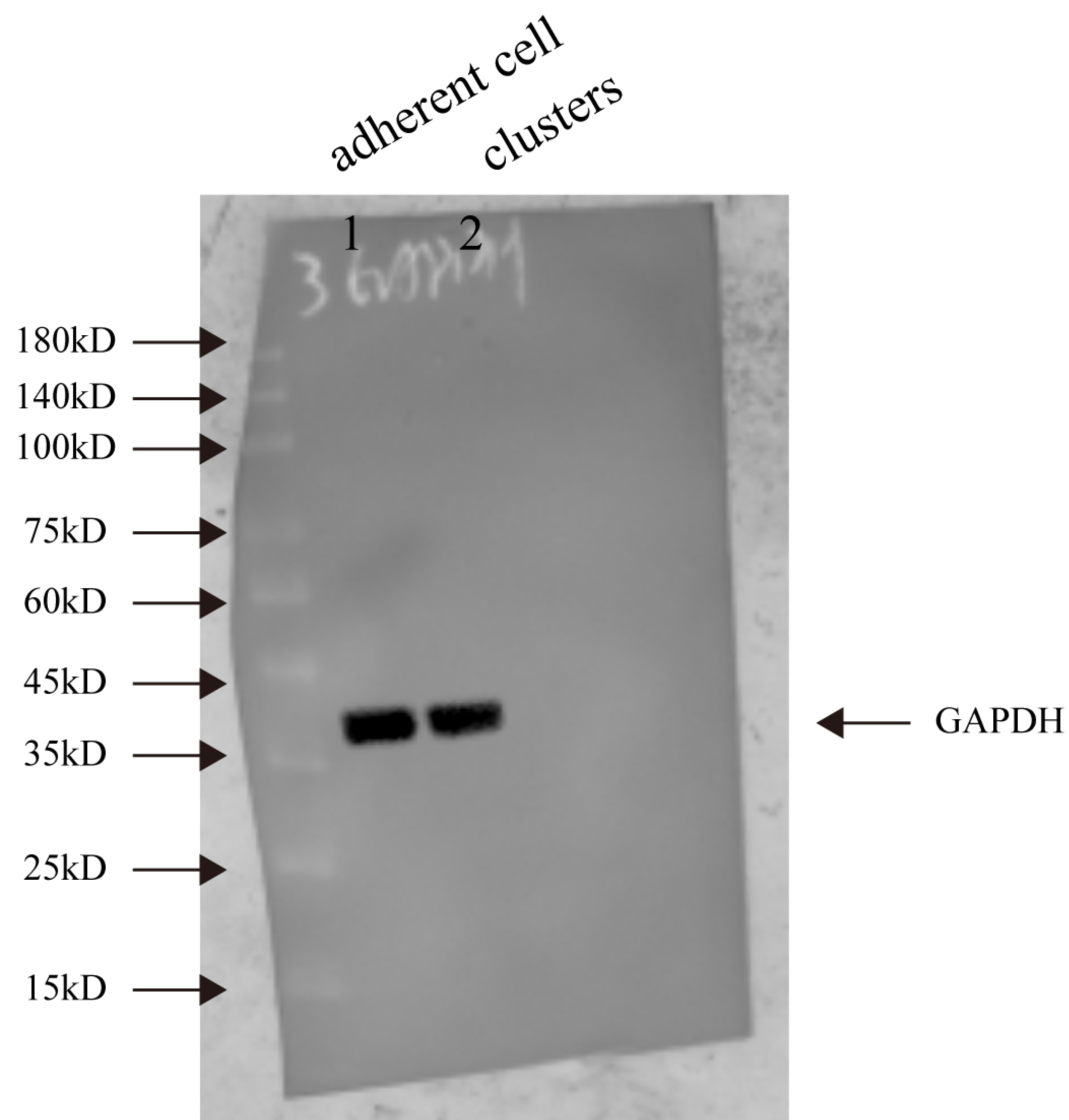

figure S2

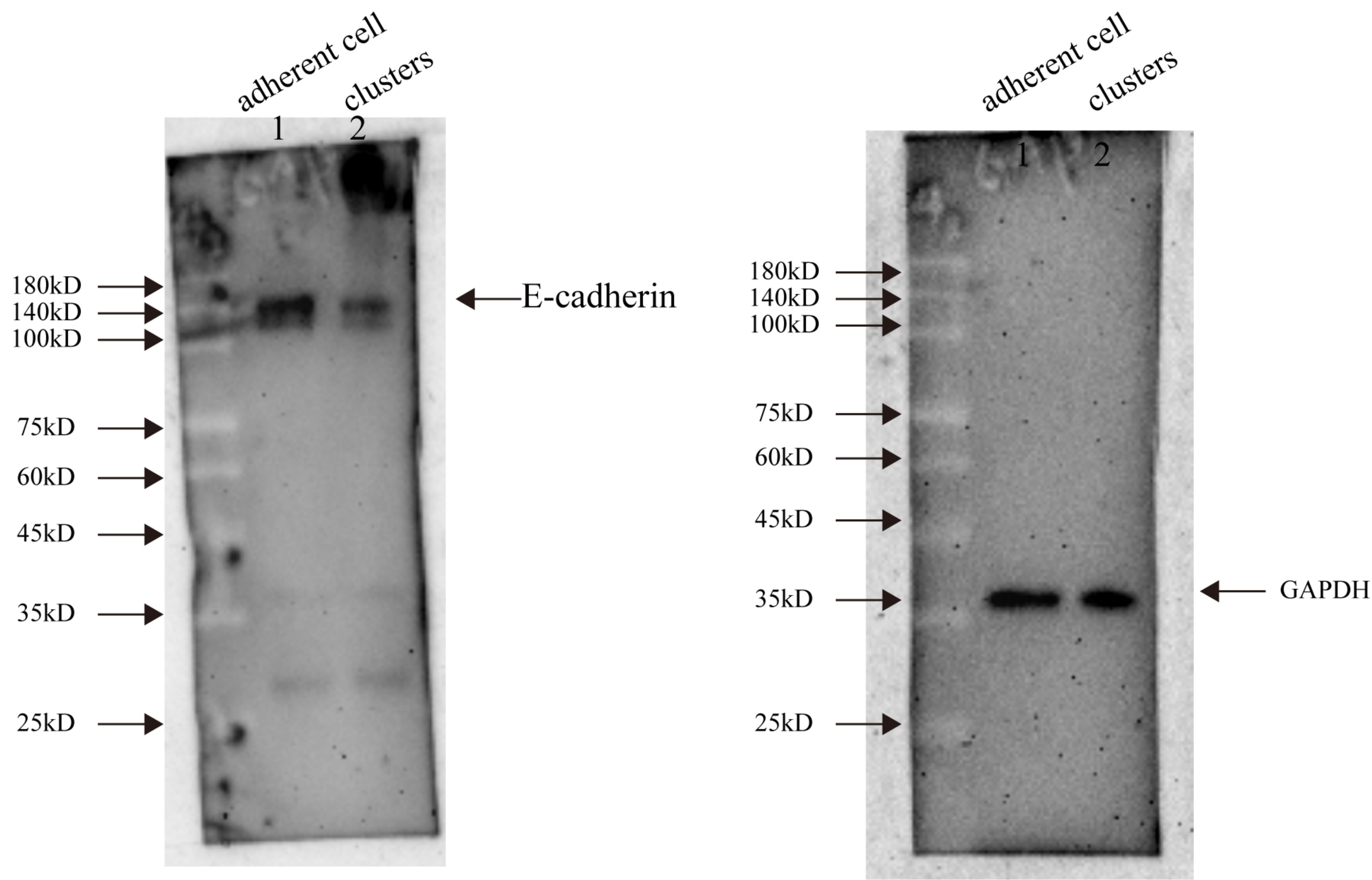

Supplement: Supplementary file 4 — Additional file 4. [file 12885_2024_12214_MOESM4_ESM.pdf]
